# Diabetes cardiomyopathy: targeted regulation of mitochondrial dysfunction and therapeutic potential of plant secondary metabolites

**DOI:** 10.3389/fphar.2024.1401961

**Published:** 2024-07-09

**Authors:** Xianglong Pan, Erwei Hao, Fan Zhang, Wei Wei, Zhengcai Du, Guangli Yan, Xijun Wang, Jiagang Deng, Xiaotao Hou

**Affiliations:** ^1^ Department of Pharmaceutical, Heilongjiang University of Chinese Medicine, Harbin, Heilongjiang, China; ^2^ Guangxi Key Laboratory of Efficacy Study on Chinese Materia Medica, Guangxi University of Chinese Medicine, Nanning, Guangxi, China; ^3^ Guangxi Collaborative Innovation Center for Research on Functional Ingredients of Agricultural Residues, Guangxi University of Chinese Medicine, Nanning, Guangxi, China; ^4^ Guangxi Key Laboratory of TCM Formulas Theory and Transformation for Damp Diseases, Guangxi University of Chinese Medicine, Nanning, Guangxi, China

**Keywords:** diabetic cardiomyopathy, mitochondrial dynamics, oxidative stress, mitophagy, insulin resistance, plant secondary metabolites

## Abstract

Diabetic cardiomyopathy (DCM) is a specific heart condition in diabetic patients, which is a major cause of heart failure and significantly affects quality of life. DCM is manifested as abnormal cardiac structure and function in the absence of ischaemic or hypertensive heart disease in individuals with diabetes. Although the development of DCM involves multiple pathological mechanisms, mitochondrial dysfunction is considered to play a crucial role. The regulatory mechanisms of mitochondrial dysfunction mainly include mitochondrial dynamics, oxidative stress, calcium handling, uncoupling, biogenesis, mitophagy, and insulin signaling. Targeting mitochondrial function in the treatment of DCM has attracted increasing attention. Studies have shown that plant secondary metabolites contribute to improving mitochondrial function and alleviating the development of DCM. This review outlines the role of mitochondrial dysfunction in the pathogenesis of DCM and discusses the regulatory mechanism for mitochondrial dysfunction. In addition, it also summarizes treatment strategies based on plant secondary metabolites. These strategies targeting the treatment of mitochondrial dysfunction may help prevent and treat DCM.

## Graphical Abstract

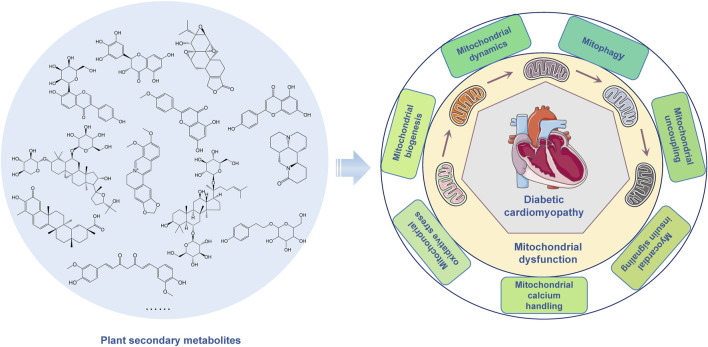

## 1 Introduction

Diabetes mellitus (DM) is a worldwide public health problem. According to the latest data of the International Diabetes Federation (IDF) in 2021, diabetes has become a health burden affecting 537 million people worldwide. It is estimated that this number will increase to 783 million by 2045 (Saeedi et al., 2019; Sun et al., 2022). The DM can be divided into type 1 (T1DM) and type 2 (T2DM), of which T2DM accounts for more than 90% of human diabetes population. Patients with T1DM or T2DM have a high risk of developing diabetes cardiomyopathy (DCM) and even heart failure (Lam, 2015). DCM is a myocardial-specific microvascular complication that results in structural and functional abnormalities of the heart muscle in diabetic patients without other cardiac risk factors such as coronary artery disease, hypertension, and severe valve disease (Heather et al., 2022). It has been estimated that the prevalence of DCM in the general population is ∼1.1%, and that it is ∼16.9% in diabetics (Rajbhandari et al., 2021). Studies have shown that hyperinsulinemia, insulin resistance, and hyperglycemia are the starting points of the cascade reaction of cardiac dysfunction in DCM (El Hayek et al., 2021; Avagimyan et al., 2022). In the state of high glucose, multiple metabolic pathways are activated and interact with each other, leading to myocardial fibrosis and hypertrophy, cardiomyocyte apoptosis, reduced coronary microcirculation perfusion, and then evolving into diastolic and systolic dysfunction and eventually diabetic heart failure (Schilling, 2015). Furthermore, there are some pathophysiological differences in the triggering of DCM between T1DM and T2DM. Patients with T1DM experience severe insulin deficiency due to autoimmune disease, leading to hyperglycemia and abnormal metabolism and function of myocardial cells. The delayed absorption of calcium in the sarcoplasmic reticulum is associated with hyperglycemia, resulting in impaired left ventricular contractile function, making contractile dysfunction symptoms more typical in T1DM patients. In contrast, T2DM is mainly caused by hyperinsulinemia and insulin resistance. Its clinical manifestations are related to myocardial fibrosis and left ventricular remodeling, leading to increased wall hardness, reduced compliance, and early induction of diastolic myocardial dysfunction in the disease (Nathan, 2015; Waddingham et al., 2015; Prandi et al., 2023).

Mitochondria are important organelles in cells, mainly responsible for the generation of cellular energy. Through the process of oxidative phosphorylation, mitochondria produce adenosine triphosphate (ATP), which provides energy for the normal physiological activities of the cell (Wang et al., 2020). In DCM, mitochondrial dysfunction has a significant impact on heart function. Due to hyperglycemia and insulin resistance, the mitochondrial function in the myocardial cells of diabetic patients may be impaired. Mitochondrial dysfunction leads to reduced ATP production, increased oxidative stress, disrupted calcium ion balance, and subsequently affects the normal function of myocardial cells. Further damage may lead to decreased myocardial contractile force, abnormal cardiac structure and function, ultimately resulting in serious consequences such as heart failure (Zhu et al., 2021). The reasons for hyperglycemia levels leading to mitochondrial dysfunction may be related to the generation of glycation end products, oxidative stress, and abnormal lipid metabolism. Hyperglycemia levels may increase the generation of glycation end products, which can bind to proteins and DNA inside the mitochondria, forming highly pathogenic crosslinks, damaging the structure and function of the mitochondria. Hyperglycemia levels may increase the level of oxidative stress inside the mitochondria, leading to an imbalance in the mitochondrial redox balance, thereby damaging the integrity of the mitochondrial membrane and the function of the electron transport chain, affecting the production of energy in the mitochondria. Additionally, hyperglycemia levels may also cause abnormal lipid metabolism, increasing the burden of lipid oxidation inside the mitochondria, leading to an increase in lipid peroxidation reactions inside the mitochondria, damaging the membrane structure and function of the mitochondria. The treatment strategy for mitochondrial dysfunction in DCM is one of the hot spots of current research (Wang et al., 2020; Wang et al., 2020). Some studies have shown that plant secondary metabolites have the potential to improve mitochondrial function and alleviate the development of DCM (Gao et al., 2022; Sodeinde et al., 2023). The plant secondary metabolites may improve mitochondrial function by regulating mitochondrial dynamics, reducing oxidative stress, maintaining calcium ion balance, promoting mitochondrial biogenesis, inducing mitophagy, inhibiting mitochondrial uncoupling, and regulating myocardial insulin signaling. Therefore, in-depth research on the mechanisms of mitochondrial dysfunction in DCM and the development of treatment strategies targeting mitochondrial function is of great significance for improving the prognosis of DCM.

## 2 The role of mitochondrial dysfunction in the pathogenesis of diabetic cardiomyopathy

The pathogenesis of DCM is complex, with early manifestations of myocardial fibrosis, functional remodeling, and associated diastolic dysfunction, progressing to systolic dysfunction, and ultimately leading to heart failure with reduced ejection fraction (EF) values. The diagnostic criteria for DCM include left ventricular diastolic dysfunction and/or reduced left ventricular ejection fraction (LVEF), left ventricular hypertrophy, and interstitial fibrosis, which can be classified as early, late, and end stages (Joubert et al., 2019). Current research indicates that the development of DCM is associated with mitochondrial dysfunction, abnormal glucose and lipid metabolism, oxidative stress, inflammation, and myocardial fibrosis. Among them, mitochondrial dysfunction plays a crucial role in the pathogenesis of DCM (Sangwung et al., 2020). Myocardial mitochondrial dysfunction refers to the accumulation of reactive oxygen species (ROS) in cells induced by factors such as ischemia or hypoxia, resulting in abnormal mitochondrial structure and function (Bozi et al., 2020). In addition, the consequence of mitochondrial dysfunction is the excessive production of ROS in the respiratory chain. When the accumulation of ROS exceeds its limit and is beyond the clearance capacity of the antioxidant system, it promotes cellular oxidative stress and induces tissue damage. Specific factors leading to excessive ROS production include hyperglycemia, hyperlipidemia, inflammatory responses, and more. These factors disrupt the electron transport chain within the mitochondria, leading to increased ROS generation. Excessive ROS negatively impacts cell structure and function, such as oxidizing lipids, proteins, and DNA, damaging cell membranes, mitochondrial membranes, and organelle structures, resulting in cell apoptosis and inflammatory reactions (Dubois et al., 2020). Additionally, mitochondrial dysfunction is often observed in the rat model of streptozotocin (STZ) induced diabetes, which is manifested in the imbalance of mitochondrial structure, the reduction of mitochondrial DNA and the reduction of the level of biologically related messenger RNA, leading to the damage of mitochondrial biology (Marciniak et al., 2014).

Mitochondria are double-membrane organelles that maintain a highly dynamic and multifunctional network (Iannetti et al., 2015). Maintaining the integrity and function of mitochondria is crucial for cellular physiology, especially in the energy-demanding heart. Within cardiac muscle cells, glucose is oxidized to ATP, which is the main source of cellular energy. The oxidation process mainly occurs within mitochondria, and through the catalytic action of enzymes such as adenosine triphosphate synthase, glucose is gradually broken down and energy is released. The ATP produced provides contraction and relaxation to myocardial cells, thereby maintaining normal heart function (Cree et al., 2017). However, in patients with diabetes, due to insufficient insulin secretion or insulin resistance, glucose cannot be effectively used, and the energy source of the heart turns to fatty acid oxidation (FAO). Over time, long-term dependence on FAO can lead to the accumulation of lipid metabolites in myocardial cells, ultimately leading to mitochondrial dysfunction and cardiac dysfunction (Croston et al., 2014). In summary, mitochondria are not only the main metabolic organelles in cells, but also important regulatory factors for improving insulin resistance. Their dysfunction plays a particularly important role in the development of DCM.

## 3 Regulatory mechanism of mitochondrial dysfunction in diabetic cardiomyopathy

### 3.1 Mitochondrial dynamics

Mammalian mitochondria are dynamic organelles with two membranes, constantly changing in length, size, quantity, and shape within the cell (Chang et al., 2022). Mitochondrial dynamics consists of mitochondrial fusion and fission, where fusion is the integration of substances from different mitochondria, while fission is the separation of mitochondria from intact parents (Sygitowicz et al., 2022). The main regulatory factors responsible for mitochondrial fusion in mammalian cells are mitochondrial fusion proteins 1 and 2 (Mfn1/2), located on the outer mitochondrial membrane (OMM), and optic atrophy 1 (Opa1), a protein located on the inner mitochondrial membrane (IMM) (Parra et al., 2011). Mitochondrial fusion is primarily divided into two steps: OMM and IMM fusion. OMM fusion is mainly mediated by Mfn1 and Mfn2, which can form bridges on the outer membrane of mitochondria and promote the outer membrane fusion of two mitochondria. The function of Mfn1 and Mfn2 is to guide the outer membranes of two mitochondria into contact with each other, and then promote the fusion of the outer membranes (Yu et al., 2020; Casellas et al., 2021). IMM fusion is mainly mediated by Opa1 protein. The Opa1 protein forms complexes on the mitochondrial inner membrane, promoting the fusion of the inner membranes of two mitochondria into one. The function of Opa1 is to facilitate membrane fusion, thereby forming a continuous inner membrane structure (Gilkerson et al., 2021; Tokuyama et al., 2023). Mitochondrial fission is the process of dividing intact mitochondrial progenitors into two or more mitochondria, leading to the redistribution of mitochondrial genetic material, structure, and quantity within the cell (Fröhlich et al., 2013). Mitochondrial fission mainly involves dynamin-related protein 1 (Drp1), fission protein 1 (Fis1), and mitochondrial fission factor (Mff) (Yang et al., 2022). Drp1 is a cytoplasmic GTP-dependent driving protein involved in the division process. Drp1 functions by localizing to the OMM, forming a ring around the mitochondria, and then hydrolyzing GTP to cause the ring to contract, resulting in mitochondrial fission. In addition, various post-translational modifications, including phosphorylation, ubiquitination, S-nitrosylation, and acetylation, control the transport of Drp1 from the cytoplasm to the mitochondria. These translation modifications promote mitochondrial fission by enhancing Drp1 oligomerization and its receptor attachment (Jin et al., 2021). Mff and Fis1 can also affect Drp1-induced mitochondrial fission through post-translational phosphorylation (Wang et al., 2022). In DCM, the loss of fusion and fission-related proteins can lead to DCM. Studies have shown that during the development of DCM, Drp1, Mff, and Fis1 are significantly upregulated in myocardial cells, while Opa1 and Mfn/2 are significantly downregulated (Ikeda et al., 2015).

Based on the essential role of mitochondrial dynamics in regulating DCM, some targeted drugs that normalize mitochondrial dynamics are used to treat hyperglycemia induced myocardial injury. Melatonin is an anti-diabetic drug. *In vivo*, it can prevent the occurrence of heart dysfunction in diabetes by inhibiting the mitochondrial fission induced by Drp1. Specifically, melatonin intervention reduces the expression level of Drp1, inhibits mitochondrial ragmentation, suppress oxidative stress, and reduces cardiomyocyte apoptosis by inhibiting SIRT1/PGC-1α dependent mitochondrial fission, thereby improving the mitochondrial function and cardiac function (Ding et al., 2018). Moreover, Mdivi-1 intervention inhibited the metastasis and translocation of Drp1, thus reducing the myocardial infarction area of STZ induced diabetes mice after ischemia reperfusion injury surgery. Mdivi-1 is considered as a Drp1 inhibitor (Ding et al., 2017). In diabetic hearts, mitochondrial fusion promoter M1 significantly increases the expression of mitochondrial fusion and Opa1, while reducing myocardial oxidative stress and improving myocardial fibrosis (Ding et al., 2020; Feng et al., 2021). Equally, dapagliflozin can promote mitochondrial fusion and inhibit fission, accompanied by prolonged cardiac action potential and stable Δψm, which may be due to upregulation of Mfn2 expression (Durak et al., 2018). Nicotinamide riboside (NR) activates SIRT1/PGC-1α/PPARα signaling transduction increases Mfn2 expression and promotes mitochondrial fusion in diabetic db/db mouse, reduces cell apoptosis, and improves heart function (Hu et al., 2022).

### 3.2 Mitochondrial biogenesis

Mitochondrial biogenesis is a process that maintains the quantity of mitochondria through regeneration, aiming to produce new and healthy mitochondria. The process of mitochondrial biogenesis is complex, mainly regulated by mitochondrial genes (mtDNA) and nuclear genes (nDNA). The mtDNA encode some of the proteins and RNA in the mitochondrial inner membrane, including important protein subunits and tRNA, while most mitochondrial proteins and other components are encoded by nDNA (Cameron et al., 2016; Tao et al., 2022). Under normal conditions, mitochondrial biogenesis enhances mitochondrial oxidative phosphorylation capacity, reduces pathological oxidative stress, maintains normal mitochondrial physiological function, and meets the energy metabolism needs of the cell. However, when the process of mitochondrial biogenesis is disrupted by exogenous or endogenous factors, it can promote mitochondrial dysfunction, leading to excessive ROS production, causing mitochondrial oxidative stress and calcium overload, thereby triggering cell apoptosis or disrupting cellular homeostasis (Bruggisser et al., 2017). PGC-1α is considered a key central mediator regulating mitochondrial biogenesis, and its expression is regulated by various upstream stimuli and post-translational modifications (Novakova et al., 2022). The expression of PGC-1α is regulated by the activation of transcription factors that act on mtDNA, consisting of sirtuin 1 (SIRT1), myocyte enhancer factor 2 (MEF2), forehead box class-O1 (FoxO1), as well as other signal inducers such as AMPK, AKT-eNOs, and calmodulin dependent protein kinase IV (CaMK IV) (Gleyzer and Scarpulla, 2016; Wang et al., 2022). PGC-1α expression is regulated by post-translational modifications, consist of methylation, acetylation, ubiquitination, and phosphorylation. In addition, PGC-1α collaborating with a series of nDNA transcription factors to regulate downstream signaling pathways, consisting of nuclear respiratory factor 1/2 (Nrf1/2), transcription factor A mitochondrial (TFAM), estrogen related receptors α (ERR-α) and PPARs (Ploumi et al., 2017). PGC-1α promotes mtDNA replication and enhances mitochondrial biogenesis by interacting with its upstream and downstream factors, which is crucial for maintaining the normal physiological function of tissues with high energy metabolism demands. AMPK is a key pathway in energy metabolism, and it is activated when the intracellular ATP level decreases or the intracellular AMP/ATP ratio increases. Activated AMPK can directly phosphorylate PGC-1α, thereby increasing its transcriptional activity. Meanwhile, AMPK can phosphorylate the threonine site 177 and serine site 538 of the PGC-1α promoter, promoting the expression of PGC-1α, a series of mitochondrial target genes, and oxidative metabolism related genes (Fernandez et al., 2011). SIRT1 is a deacetylase that regulates gene expression and metabolic processes within cells, particularly playing a crucial role in energy metabolism and oxidative stress. SIRT1 can increase its expression by deacetylating PGC-1α, thereby promoting mitochondrial biogenesis (Wu et al., 2023). Some evidence has suggested an association between mitochondrial biogenesis and DCM. Diao et al. found reduced mtDNA replication and transcription, damaged mitochondrial ultrastructure, downregulation of PGC-1α, leading to impaired mitochondrial biogenesis and cardiac injury in a DCM rat model (Diao et al., 2021). Research by Tao et al. has shown that MiR-144 is downregulated in HG-induced myocardial cells and STZ-induced DCM rats. Overexpression of MiR-144 enhances mitochondrial biogenesis and inhibits cell apoptosis, while inhibiting MiR-144 shows the opposite results. In addition, Rac-1 has been identified as a regulatory gene of MiR-144. Reduced expression of Rac-1 activates AMPK phosphorylation and PGC-1α deacetylation, leading to increased mitochondrial biogenesis and reduced cell apoptosis (Tao et al., 2020). Adiponectin (APN), as an upstream activator of AMPK, showed a significant decrease in plasma levels of APN in ob/ob mouse. The researchers further validated the hypothesis that there is a causal relationship between APN reduction and mitochondrial biogenic damage. After 1 week of APN treatment in ob/ob mice, activating AMPK and reducing PGC-1α acetylation can increase mitochondrial biogenesis and alleviate mitochondrial diseases. On the contrary, knocking out APN inhibits AMPK/PGC-1α signaling and impairs mitochondrial biogenesis (Yan et al., 2013). Tetrahydrobiopterin (BH4) is a novel endogenous activator of CaMKK2 that can participate in regulating vascular and cardiac function. Research has shown that in the db/db mouse model lacking BH4, ROS production increases and induces mitochondrial dysfunction. Supplementing BH4 can improve cardiac function, correct myocardial morphological abnormalities, and increase mitochondrial biogenesis by activating the CaMKK2/PGC-1α signaling pathway (Kim et al., 2020). Antioxidant pterostilbene in blueberries regulates AMPK/NRF2/HO-1/PGC-1α signal transduction, which can reduce oxidative stress and inflammation and improve mitochondrial biogenesis in a high glucose rat model (Kosuru et al., 2018). In conclusion, an increasing amount of evidence suggests that abnormal mitochondrial biogenesis is a major factor leading to DCM, and regulating the process of mitochondrial biogenesis has become a potential strategy for treating DCM.

### 3.3 Mitophagy

**FIGURE 1:**
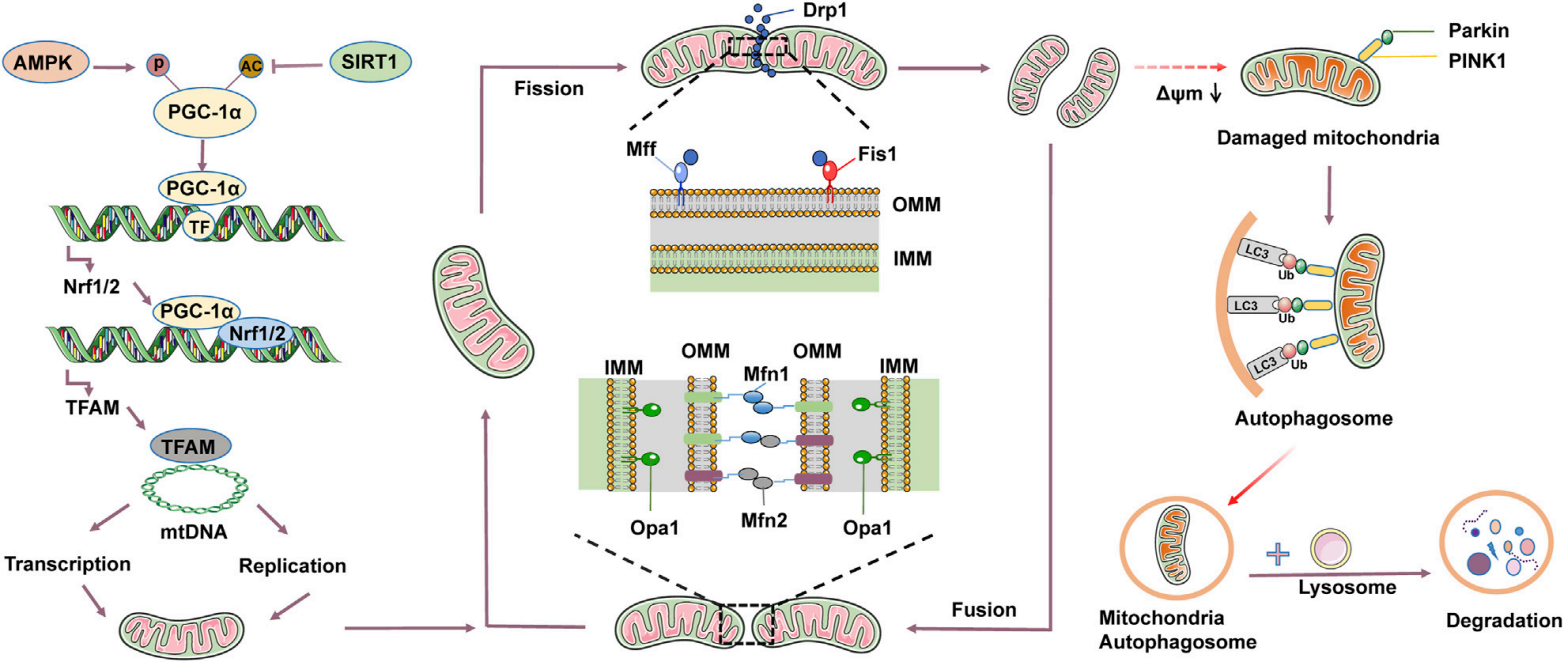
Mitochondrial quality control mainly includes mitochondrial dynamics, mitophagy, and mitochondrial biogenesis. Mitochondrial dynamics involve fusion and fission. Mitochondrial fusion is induced by homotypic and heterotypic interactions between mitochondrial fusion proteins 1 and 2 (Mfn1/2) of the outer mitochondrial membrane (OMM) and optic atrophy 1 (Opa1) of the inner mitochondrial membrane (IMM). Mitochondrial fission mainly involves dynamin-related protein 1 (Drp1), mitochondrial fission factor (Mff), and mitochondrial fission 1 (Fis1). Drp1 is recruited to OMM through interactions with Fis1 and Mff. Under physiological conditions, mitochondrial fusion and fission constraint each other to reach mitochondrial dynamic equilibrium. Mitochondrial fission contributes to the clearance of damaged or aging mitochondria, leading to a decrease in mitochondrial membrane potential (Δψm), thereby activating mitophagy. Mitophagy mainly consists of 4 key steps: 1) Depolarization of damaged mitochondria, leading to the loss of membrane potential. 2) Enclosure of mitochondria by autophagosome to form mitochondria autophagosome. 3) Fusion of mitochondria autophagosome with lysosomes. 4) Degradation of mitochondrial contents by lysosomes. Mitochondrial biogenesis is a tightly regulated process. Adenosine monophosphate-activated protein kinase (AMPK) can phosphorylate peroxisome proliferator-activated receptor-γ coactivator-1α (PGC-1α), while sirtuin 1 (SIRT1) can acetylate PGC-1α. PGC-1α activates signaling molecules such as nuclear respiratory factor 1/2 (Nrf1/2) and transcription factor A mitochondrial (TFAM), driving the replication and transcription of mtDNA, and translating into proteins, assembling to form new mitochondria.

Mitophagy contributes to maintaining the health and function of mitochondria within cells, which is crucial for cellular metabolism and survival. The process of mitophagy involves the selective targeting of autophagosomes to phagocytize dysfunctional or damaged mitochondria, which are then transferred to lysosomes for degradation (Li et al., 2021; Saito et al., 2021). There are currently three pathways that induce mitophagy, consist of the phosphatase and tensin homologue-induced putative kinase 1 (PINK1)/Parkin pathway, the FUN14 domain-containing (FUNDC1) pathway, and the BCL2/adenovirus E1B 19kDa interacting protein 3 (BNIP3)/NIX pathway (Tong et al., 2021). Among them, the most studied mitophagy pathway is PINK1/Parkin (Andres et al., 2017). PINK1, a serine/threonine kinase, functions as a messenger to relay the collapse of Δψm to Parkin. Usually, PINK1 is swiftly transported to the mitochondrial matrix and cleaved by mitochondrial proteases (Jin et al., 2010). Therefore, under normal circumstances, the content of PINK1 in mitochondria is relatively low. However, when mitochondria are damaged, the decrease in Δψm is directly related to the increase in PINK1 on OMM. PINK1 and Parkin jointly control the removal of damaged mitochondria (Zhang et al., 2020). Similarly, Parkin acts as an E3 ubiquitin ligase and remains cytosolic in normal conditions. However, upon depolarization of the mitochondrial membrane, it rapidly translocates to the OMM and ubiquitinates proteins located in the outer membrane, thereby marking them for elimination (Cai et al., 2022). Currently, it has been found that many Parkin substrates accumulate on OMM, such as Mfn1/2, OMM transporters, and voltage dependent anion channels (Poole et al., 2010; Morciano et al., 2020). In DCM, mitophagy enhances the regeneration of cardiomyocyte mitochondria and stimulates biogenesis, which can normalize the morphology and bioenergy of cardiac mitochondria (Wang et al., 2018; Wang et al., 2019). In addition, mitophagy also reduced lipid accumulation, improved mitochondrial homeostasis, and restored the diastolic and systolic functions of diabetes heart (Zhou et al., 2019). Tong et al. used the mito-Keima method to evaluate mitophagy in the GFP-LC3 mouse myocardial cell model induced by a high-fat diet (HFD). Knocking out Parkin inhibits mitophagy, increases lipid accumulation, and exacerbates diastolic dysfunction. However, injection of Tat-Beclin1 (TB1) can activate mitophagy, reduce lipid accumulation, and prevent diastolic dysfunction in the heart. The study suggests that inhibiting mitophagy leads to mitochondrial dysfunction and lipid accumulation, thereby exacerbating diabetic cardiomyopathy. In contrast, the activation of mitophagy can prevent HFD-induced diabetic cardiomyopathy (Tong et al., 2019). Alisporivir is a non immunosuppressive cyclosporin derivative and a selective inhibitor of mitochondrial permeability transition pore (mPTP). Belosludtseva et al. treated HFD combined with STZ-induced diabetic mice with Alisporivir (2.5 mg/kg/d) for 20 days. Alisporivir improved mitochondrial swelling and ultrastructural changes in the myocardial cells of diabetic mice, increased the mRNA expression levels of Pink1 and Parkin in the heart tissue, and reduced the accumulation of lipid peroxides. The study suggests that Alisporivir can exert a protective effect on the heart by inducing mitophagy (Belosludtseva et al., 2021). Therefore, pharmacological methods of targeting mitophagy may be effective treatment methods to slow down the progression of DCM and improve prognosis (Figure 1).

### 3.4 Mitochondrial oxidative stress

Diabetic cardiomyopathy is a disease characterized by structural and functional abnormalities of the myocardium caused by hyperglycemia, and its pathogenesis is closely related to mitochondrial oxidative stress. During the process of mitochondrial oxidative phosphorylation (OXPHOS), NADH and FADH2 serve as electron donors, releasing electrons at electron transport chain (ETC) complexes I and II respectively. These electrons travel from complexes I and II through ubiquinone to complex III, and then in complex IV, O_2_ combines with the electrons transferred from complex III by cytochrome C, generating H_2_O. Protons are sequentially transferred to the intermembrane space through complexes I, III, and IV. Ultimately, the protons in the intermembrane space are transported back to the mitochondrial matrix through complex V, generating ATP. It is worth noting that electrons can easily leak from complexes I and III, leading to the generation of ROS (Teshima et al., 2014; Jia et al., 2016). In the environment of insulin resistance and hyperglycemia, the mitochondrial OXPHOS process is impaired, leading to reduced ATP synthesis and the production of a large amount of ROS. ROS is a highly active molecule that can cause oxidative damage by interacting with proteins, lipids, DNA, and other molecules inside the mitochondria, leading to oxidative stress. Oxidative stress affects the structure and function of mitochondria, thereby influencing cellular energy metabolism and signal transduction. In addition, oxidative stress also activates some proteins on the OMM, such as Bax and Bcl-2, which are involved in regulating cell apoptosis (Quan et al., 2020; Dewanjee et al., 2021).

Link between mitochondria oxidative stress and lipotoxicity. The augmented uptake of fatty acids (FA) by mitochondria and subsequent oxidation in diabetic cardiac tissues may surpass the respiratory capacity of mitochondria, leading to the buildup of harmful lipid metabolites. This accumulation can result in cardiac lipotoxicity and impairment of mitochondrial function (Jia et al., 2018). Adenosine monophosphate-activated protein kinase (AMPK) typically enhances the generation of new mitochondria by activating peroxisome proliferator-activated receptor-γ (PPAR-γ) coactivator-1α (PGC-1α), a key metabolic regulator of mitochondrial biogenesis and respiratory performance (Crisafulli et al., 2020). The impairment of the AMPK/PGC-1α signalling pathways associated with FAO occurs during the advanced stage of DCM, thereby exacerbating mitochondrial dysfunction (Nakamura et al., 2022). Additionally, an increase in FAO can promote the production of ROS and induce cardiac oxidative stress and inflammation. The elevated ROS levels further contribute to mitochondrial dysfunction, leading to lipid accumulation, fibrosis, diastolic dysfunction, and ultimately exacerbating heart failure (Murtaza et al., 2019). Similarly, an increase free fatty acids (FFAs) in the blood can lead to an increase of FA in cardiomyocytes. Excessive FA accumulate in cells in the form of lipid droplets and triglycerides, while diacylglycerol and ceramide also increase (Nakamura et al., 2020). Diacylglycerol triggers the exacerbation of insulin resistance and oxidative stress by activating protein kinase C (PKC). Research has demonstrated that diacylglycerol serves as a toxic lipid intermediate in cardiac tissue (Chokshi et al., 2012). The accumulation of ceramide leads to a substantial production of mitochondrial ROS, which induces mitochondrial dysfunction and oxidative stress within myocardial mitochondria (Law et al., 2018; Kim et al., 2020). Moreover, the anti-diabetic drug empagliflozin (SGLT2 inhibitor) can lead to a decrease in plasma volume and cardiac preload, regulate superoxide dismutase (SOD) levels and lipid metabolism, reduce oxidative stress, improve mitochondrial function, and thus play a protective effect on the heart (Kaludercic et al., 2020).

Recent research has highlighted the close relationship between ferroptosis and mitochondrial oxidative damage. Research shows that abnormal mitochondrial ferroptosis occurs in the heart of diabetes mice, which is mainly manifested by the decrease of Δψm, the downregulation of the expression of SOD and glutathione peroxidase 1 (GPX 1) in mitochondria, and the significant increase in mitochondrial ROS levels (Fang et al., 2020). Furthermore, another study has demonstrated that feeding mice a high-iron diet leads to severe myocardial damage, manifested as iron overload, increased lipid peroxidation, and decreased glutathione levels (Sampaio et al., 2014). Du et al. treated STZ-induced diabetic C57BL/6J mice with canagliflozin for 6 weeks, as well as H_9_C_2_ cardiomyocytes induced with high glucose (HG) for 24 h. Their *in vivo* and *in vitro* studies showed that canagliflozin inhibits the deposition of total iron and Fe^2+^, downregulates the expression of ferritin heavy chain (FTN-H), upregulates the cystine-glutamate antiporter (xCT), increases the level of Δψm in the myocardium, reduces ROS levels, and inhibits mitochondrial oxidative damage. This exerts a cardioprotective effect by inhibiting ferroptosis (Du et al., 2022). Ferroptosis is a novel form of programmed cell death, and more clinical research is needed to support its role in the prevention and treatment of diabetic cardiomyopathy. In conclusion, targeting ferroptosis may provide a new strategy for the prevention and treatment of DCM.

### 3.5 Mitochondrial uncoupling

Mitochondrial uncoupling is an important physiological mechanism that can regulate energy metabolism and heat generation within cells. Under normal circumstances, there is a proton gradient inside the mitochondria, meaning there is a difference in proton concentration between the inner and outer membranes. During the process of uncoupling, the proton gradient is released to maintain the balance of proton concentration. The released protons will bind to the uncoupling proteins (UCPs) protein on the IMM, forming a channel that allows protons to pass through the mitochondrial inner membrane instead of through ATP synthase. In other words, the proton gradient will not be used to produce ATP, but will interact with UCPs to generate heat energy (Azzu et al., 2010). UCPs located on the IMM are considered the main mediators of mitochondrial uncoupling. UCPs family consists mainly of UCP1-UCP5. UCP1 is highly expressed in the mitochondria of adipose tissue and is mainly responsible for temperature regulation. UCP2 is widely expressed in most tissues, such as the myocardium, and is involved in the body’s energy metabolism. UCP3 is predominantly expressed in skeletal muscle, while UCP4 and UCP5 are present in brain tissue. The expression of different UCPs in different tissues reflects different physiological functions. Currently, research on DCM mainly focuses on UCP2 and UCP3, especially UCP2 (Mailloux and Harper, 2011; Akhmedov et al., 2015).

In DCM, UCP2 may be involved in the development of the disease through various physiological mechanisms. UCP2 can regulate the permeability of the IMM, increasing the proton permeability within the mitochondria, thereby reducing the electrochemical load of the mitochondria, decreasing the proton gradient within the mitochondria, inhibiting the coupling of the tricarboxylic acid cycle and oxidative phosphorylation, ultimately leading to mitochondrial uncoupling. This uncoupling may result in reduced ATP synthesis within the cells, while increasing the production of ROS within the mitochondria, thereby triggering oxidative stress and cell damage (Cadenas, 2018; Nirengi et al., 2020; Ho et al., 2022). In addition, UCP2 may also be involved in the occurrence of DCM by regulating lipid metabolism. Studies have shown that UCP2 can affect lipid metabolism pathways, including FAO and synthesis, thereby influencing intracellular lipid content and oxidative stress levels. The dysregulation of lipid metabolism in relation to UCP2 regulation may lead to increased lipid accumulation in myocardial cells, thereby affecting myocardial cell function (Diano et al., 2012). Due to the elevated levels of FFA in DCM, the enhanced expression of UCPs is directly induced by PPARα, thereby affecting the permeability and proton leak of IMM, inhibiting ATP production, which is typically observed in failing hearts (Wang et al., 2021). PPARα can promote the oxidative metabolism of FA, including FA uptake, transport, β-oxidation, and the permeability of the IMM. These processes directly impact the electrochemical gradient of the IMM, thus influencing the degree of mitochondrial uncoupling (Lee et al., 2017; Crescenzo et al., 2019; Liu et al., 2022). Mitochondrial uncoupling is mainly mediated by the activation of UCPs. In a report by Dludla et al., it is suggested that guanosine diphosphate (GDP) can inhibit the activation of UCPs, preventing mitochondrial proton leak in diabetic db/db mice (Dludla et al., 2018). Additionally, some studies have suggested that overexpression of UCP2 may lead to mitochondrial dysfunction and exacerbate the development of diabetic cardiomyopathy. The application of PPAR agonists (such as pioglitazone) can regulate the expression of UCP2, significantly reducing the levels of free fatty acids in the plasma of type 2 diabetic patients, increasing Δψm, and restoring normal mitochondrial function (Wassef et al., 2018). Interestingly, in another study, it was found that UCP2 was downregulated in a STZ-induced diabetic mouse model, leading to a decrease in Δψm and an increase in cell death. However, overexpression of mitochondrial aldehyde dehydrogenase (ALDH2) can reverse this situation, resulting in beneficial effects on cardiac structure and function, mitochondrial function, and cell survival (Zhang et al., 2023). In summary, targeted regulation of UCP2 will enhance our understanding of DCM.

### 3.6 Mitochondrial calcium handling

The mitochondrial calcium handling plays a crucial role in maintaining normal cellular function. Mitochondrial calcium homeostasis is the balance of intracellular calcium concentration maintained by mitochondria by regulating the uptake and release of calcium ions (Diaz et al., 2021). Within mitochondria, Ca^2+^ enhances oxidative phosphorylation activity (including mitochondrial complexes I, III, IV, and complex V), as well as activates pyruvate dehydrogenase complex (PDC), alpha-ketoglutarate dehydrogenase, and isocitrate dehydrogenase to enhance ATP regeneration (Ketenci et al., 2022). Various calcium channel proteins exist on the inner mitochondrial membrane, with the most important ones being mitochondrial calcium uniporter (MCU) and voltage-dependent anion channel (VDAC). MCU is a key protein that regulates the uptake of calcium ions on the IMM, while VDAC is involved in regulating the opening of calcium channels on the OMM. The functional abnormalities or changes in expression levels of these channel proteins may lead to excessive or insufficient uptake of calcium ions in mitochondria, thereby affecting mitochondrial function (Hamilton et al., 2021). Mitochondrial calcium uptake protein 1 (MICU1), located on the inner mitochondrial membrane, interacts with MCU to regulate the uptake of calcium ions by the mitochondria. When the intracellular calcium ion concentration increases, MICU1 binds to the MCU channel and inhibits its activity, thereby reducing the uptake of calcium ions within the mitochondria. This regulatory effect helps maintain the balance of calcium ions within the mitochondria, preventing excessive uptake of calcium ions from damaging mitochondrial function (Ji et al., 2017). In DCM, abnormal mitochondrial calcium handling may lead to mitochondrial dysfunction and damage to myocardial cells. Studies have shown that the expression level of MICU1 is downregulated in 12 week old db/db mouse cardiomyocytes, accompanied by mitochondrial dependent intrinsic apoptosis. In this mouse model, the reconstruction of MICU1 can reduce myocardial hypertrophy and fibrosis, inhibit cell apoptosis, and normalize cardiac function. Furthermore, studies have shown that upregulation of MICU1 increases mitochondrial Ca^2+^ uptake, weakens mitochondrial ROS production and cell apoptosis (Dillmann, 2019). This dysfunctional calcium handling can be rescued by restoring calcium to mitochondria, thereby enhancing mitochondrial activity and energy production. Similarly, in the STZ induced diabetes mouse model, Suarez et al. found that the heart of diabetes mice showed changes in the expression of MCU and MCU members, which led to the decrease of mitochondrial Ca^2+^ uptake, mitochondrial energy function and cardiac function. On the contrary, normalization of MCU levels based on adeno-associated viruses in these hearts restored mitochondrial Ca^2+^ homeostasis, reduced PDC phosphorylation levels, improved cardiac energy metabolism and cardiac function (Suarez et al., 2018). In addition, research has shown that GrpE-like 2 (Grpel2) can reduce myocardial ischemia/reperfusion injury by inhibiting mitochondrial calcium overload mediated by MCU. Grpel2 levels are decreased in STZ-induced DCM, and overexpression of Grpel2 can mitigate mitochondrial dysfunction and apoptosis in DCM by maintaining dihydrolipoyl succinyltransferase (DLST) input to mitochondria (Yang et al., 2023).

### 3.7 Myocardial insulin signalling

**FIGURE 2:**
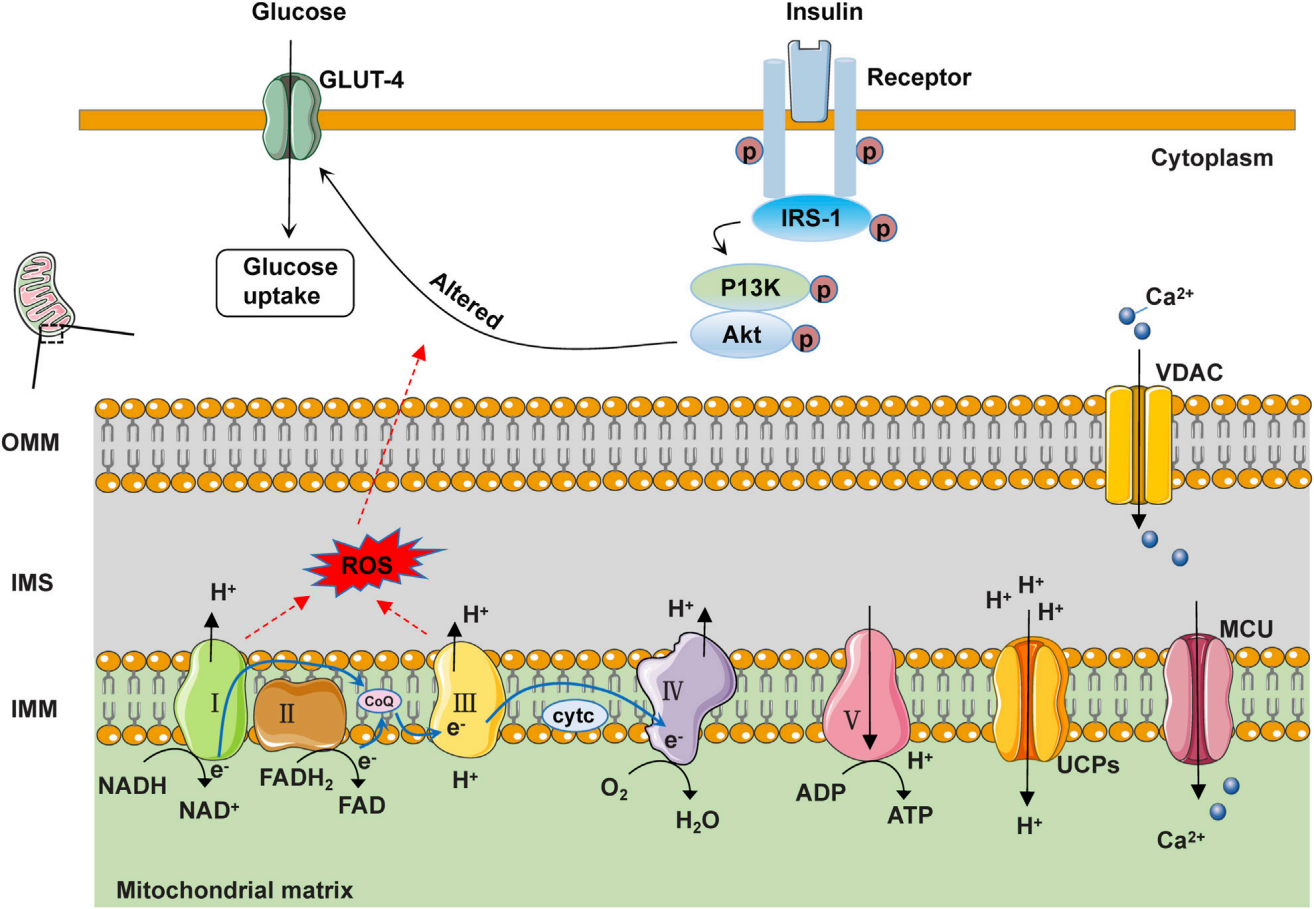
Schematic diagram of the structure and function of mitochondria under physiological conditions. In IMM, NADH and FADH2 serve as electron donors, releasing electrons at complexes I and II, respectively. These electrons pass from complexes I and II through ubiquinone to reach complex III. Subsequently, at complex IV, O2 in the matrix accepts electrons transferred from cyt c by complex III, generating H2O. Protons are sequentially transferred to the intermembrane space (IMS) through complexes I, III, and IV. Ultimately, the protons in the IMS are transported back to the matrix through complex V to generate ATP. Additionally, electrons can easily leak from complexes I and III, leading to the formation of reactive oxygen species (ROS). Some protons return to the mitochondrial matrix through uncoupling proteins (UCPs), generating heat. Free Ca^2+^ in the cytoplasm can enter the mitochondrial matrix through the voltage-dependent anion channel protein (VDAC) on the OMM and the mitochondrial calcium uniporter (MCU) on the IMM. When insulin binds to the insulin receptor, the activated receptor phosphorylates the IRS-1 protein. IRS-1 further activates the phosphorylation activity of PI3K and Akt, thereby promoting the transport of GLUT4 and glucose uptake.

Diabetic cardiomyopathy is a heart disease caused by hyperglycemia and insulin resistance. Diabetic patients often experience insulin resistance (reduced sensitivity of cells to insulin), leading to abnormal insulin signal transduction, including reduced expression and function of insulin receptor substrate-1 (IRS-1) and blocked PI3K/Akt signaling pathway. These abnormalities can affect cellular glucose uptake and utilization, leading to cellular metabolic disorders (Salvatore et al., 2021). IRS-1 is one of the key molecules in insulin signal transduction. Under normal circumstances, insulin binds to its receptor, activates IRS-1, and then activates the PI3K/Akt signaling pathway. Activated Akt can promote the translocation and transposition of GLUT4, thereby increasing glucose uptake. However, in DCM, insulin signal transduction is inhibited, leading to a decrease in the phosphorylation level of IRS-1, which affects the function of IRS-1, making it unable to effectively recruit and activate the PI3K/Akt pathway (Chen et al., 2022). This abnormal insulin signal transduction may directly affect mitochondrial function. Mitochondria are the energy production centers within cells, responsible for producing most of the intracellular ATP (Qi et al., 2013). Insulin signaling abnormalities can affect the structure and function of mitochondria, leading to changes in mitochondrial membrane permeability, disruption of oxidative phosphorylation, and increased oxidative stress. These changes may result in mitochondrial dysfunction and subsequently affect the energy metabolism of myocardial cells (Chen et al., 2022). Research has shown that knocking out the insulin receptor in the heart leads to reduced glucose uptake and increased mitochondrial ROS production in the heart (Zhou et al., 2018; Gargiulo et al., 2020). Knockout of IRS-1 reduces ATP content in myocardial cells, impairs cardiac metabolism and function, increases fibrosis, and exacerbates heart failure (Battiprolu et al., 2012; Bugger et al., 2012). The results of ventricular muscle biopsy obtained from T2DM patients have shown reduced PI3K/Akt signaling, as well as decreased GLUT4 expression and translocation (Hou et al., 2019). The E3 ubiquitin ligase mitsugumin 53 may play a crucial regulatory role in maintaining insulin signaling. Elevated levels of mitsugumin 53 in a T2DM mouse model are associated with increased degradation of insulin receptor and IRS-1 proteins. Overexpression of mitsugumin 53 inhibits insulin signaling transduction and promotes cardiac fibrosis (Song et al., 2013; Liu et al., 2015). Conversely, downregulation of mitsugumin-53 may be a potential therapeutic approach to prevent diabetic cardiomyopathy from progressing to heart failure. In addition, abnormal insulin signaling may also lead to increased apoptosis, while the activation of the mitogen-activated protein kinase (MAPK) signaling pathway may exacerbate this process. Under normal circumstances, insulin promotes cell proliferation and growth, maintains the structure and function of cardiomyocytes, further promotes glucose uptake and utilization, and provides the energy substrates needed for mitochondria by regulating the MAPK signaling pathway. However, hyperglycemia and insulin resistance can lead to increased levels of inflammatory factors and oxidative stress within cells, thereby activating the MAPK signaling pathway. The activated MAPK signaling pathway can promote cardiomyocyte apoptosis, fibrosis, and inflammatory reactions, accelerating the progression of myocardial disease (Jia et al., 2018). In general, insulin signaling transduction regulates various signaling molecules and pathways, affecting the energy metabolism and function of cells. When these signaling pathways become disrupted or imbalanced, it can lead to insulin resistance and the development of diseases such as diabetes. Therefore, a thorough understanding of the molecular mechanisms of insulin signaling transduction is helpful in revealing the pathogenesis of diabetes and providing new targets and strategies for the treatment and prevention of related diseases (Figure 2).

## 4 Potential targets and therapeutic strategies of mitochondrial dysfunction in diabetes cardiomyopathy

**FIGURE 3:**
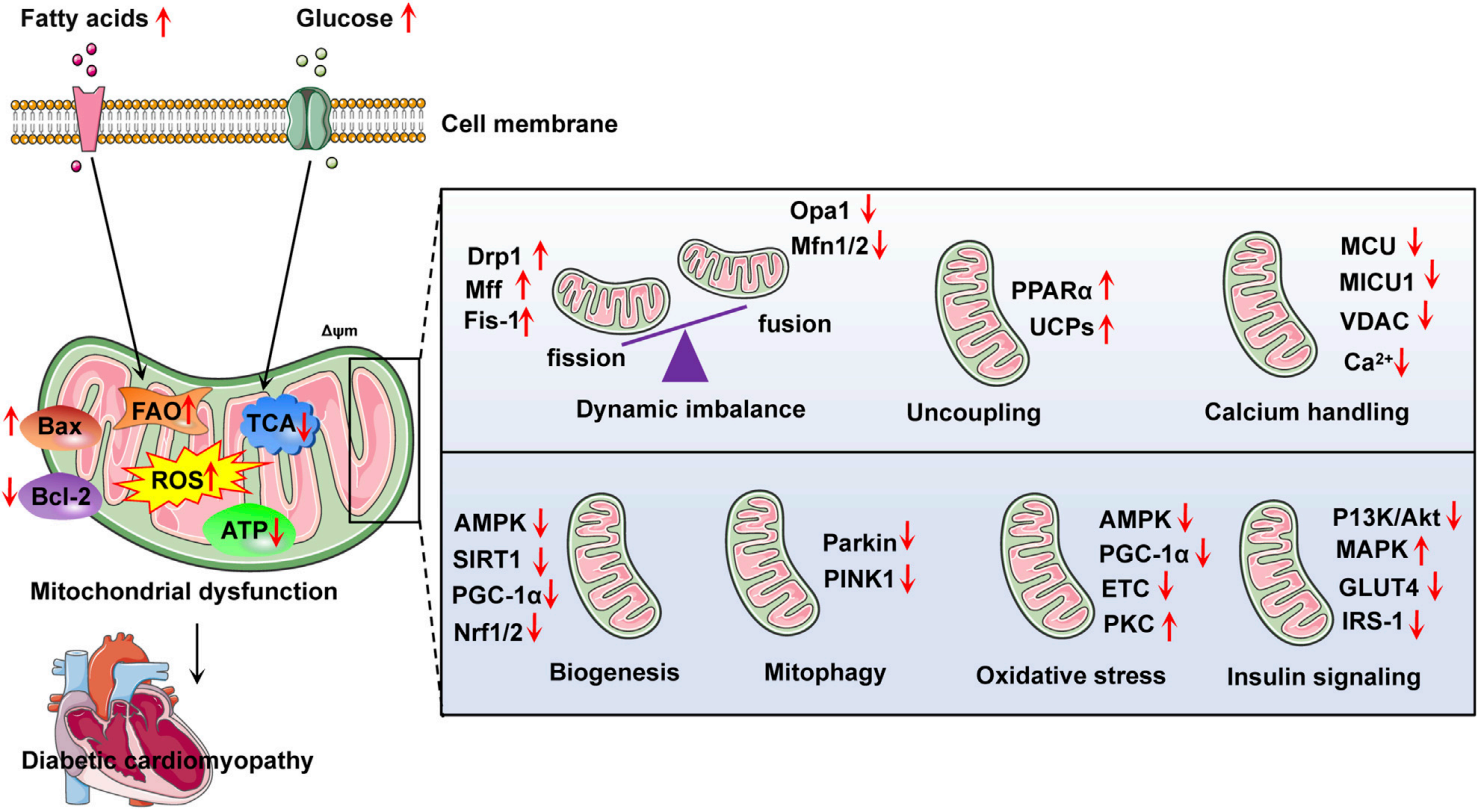
Summarizes some relevant targets in the development of diabetic cardiomyopathy with mitochondrial dysfunction. Abnormal metabolism of fatty acid and glucose may lead to mitochondrial dysfunction, thus aggravating the development of diabetes cardiomyopathy. FAO, fatty acid oxidation; ROS, reactive oxygen species; ATP, adenosine triphosphate; Bcl-2, B-cell lymphoma-2; Bax, BCL2-associated X protein; PPARα, peroxisome proliferator-activated receptor-α; MCU, mitochondrial calcium uniporter; VDAC, voltage-dependent anion channel; AMPK, adenosine monophosphate-activated protein kinase; PINK1, phosphatase and tensin homolog induced putative kinase 1; Opa1, optic atrophy 1; Mfn1/2, mitofusion 1/2; Drp1, dynamin related protein 1; UCPs, uncoupling proteins; PKC, protein kinase C; ETC., electron transport chain; IRS-1, insulin receptor substrate 1; Nrf1/2, nuclear respiratory factor 1/2; GLUT4, glucose transporter type 4; Δψm, mitochondrial membrane potential; MAPK, mitogen-activated protein kinase.

Currently, there is no effective treatment regimen to reverse or control the progression of DCM. Strict blood sugar control is the most accepted initial methods (Thai et al., 2021). After the diagnosis of DCM, cardiac function changes rapidly and the risk is high of further development into heart failure. Because mitochondrial dysfunction is the most common driving factor of diabetes cardiomyopathy, mitochondrial dynamics imbalance, excessive oxidative stress, damaged mitophagy, impaired mitochondrial biosynthesis, and impaired mitochondrial calcium processing constitute potential therapeutic targets (Varkuti et al., 2020) (Figure 3).

Some antidiabetic medications currently in use may directly or indirectly interfere mitochondrial abnormalities associated with DCM, such as metformin, dapagliflozin, and empagliflozin. Metformin is a widely used diabetes drug in clinic. Metformin promotes mitochondrial autophagy and improves myocardial cell dysfunction through AMPK-dependent or -independent mechanisms in diabetic hearts. Additionally, metformin can stimulate mitochondrial biogenesis in high glucose-induced cardiomyocytes by upregulating transcription factors related to mitochondrial biogenesis (such as PGC-1α and TFAM) (Abdel et al., 2018; Liu et al., 2020). However, the detailed mechanism by which metformin regulates DCM mitochondrial function remains unclear (Packer, 2020). In addition, for diabetic patients at risk of cardiovascular disease, cautious consideration of the use of metformin and close monitoring of cardiovascular events may be necessary. As new hypoglycemic drugs, Dapagliflozin and empagliflozin are sodium-glucose cotransporter-2 inhibitors (SGLT2is) that exhibit protective effects on reducing cardiovascular mortality and heart failure in patients with T2DM. Recent evidence suggests that SGLT2is may play a protective role in the heart by regulating the mitochondrial function in diabetes models. In obese insulin-resistant rats induced by a HFD, administration of dapagliflozin 4 weeks before myocardial ischemia/reperfusion injury can effectively reduce mitochondrial ROS production, swelling, as well as depolarization. Studies have also shown that dapagliflozin enhances mitochondrial ultrastructure by decreasing fragmentation and ridge loss within the mitochondria (Wiviott et al., 2019; Li and Zhou, 2020). Additionally, in STZ-induced diabetic rats fed with a HFD, empagliflozin has been shown to improve atrial structural and electrical remodeling by enhancing mitochondrial respiratory function and biogenesis. It plays a crucial role in mitochondrial biogenesis by activating the PGC-1α-NRF1-TFAM signaling pathway to prevent the induction of atrial fibrillation (Shao et al., 2019). However, the exact role of mitochondrial biogenesis in the occurrence and progression of ischemic cardiomyopathy or atrial fibrillation in diabetic patients remains unclear.

Besides positive effect of antidiabetes drugs on DCM mitochondria, other strategies may also be promising treatments for mitochondrial dysfunction (Ketenci et al., 2022). For example, targeting mitochondrial ROS clearance is a positive potential therapeutic strategy in DCM. The mitochondrial targeted drugs Mn (III) tetrakis (4-benzoic acid) porphyrin (MnTBAP) and mitoquinone (MitoQ) have been shown to have the ability to alleviate oxidative stress in preclinical studies. MnTBAP intervention can reverse myocardial oxidative stress and improve mitochondrial bioenergy in a mouse model of metabolic syndrome. MitoQ treatment can reduce ROS accumulation and demonstrate anti-inflammatory and anti-oxidantion effects in T2DM patients (Ilkun et al., 2015; Escribano et al., 2016). In addition, targeting mitochondrial regulators may also have beneficial effects on DCM. AMPK is the main regulator of mitochondrial energy homeostasis. Activation of AMPK enhances the expression and translocation of GLUT4, enhances insulin stimulated glucose uptake, and promotes mitochondrial biogenesis. Mechanically, activated AMPK in myocardial cells increases glucose uptake and utilization, while also negatively regulating mTOR signaling and gluconeogenesis, lipid as well as protein synthesis (Abdel et al., 2017). The activation of AMPK is essential to prevent the progression of diabetes cardiomyopathy. Therefore, AMPK is considered as an effective target for drug discovery and development to prevent and reverse DCM. Moreover, PPARα plays a crucial regulatory role in mitochondrial oxidative stress and myocardial glucose and lipid metabolism. The role of PPARα in diabetes cardiomyopathy is mainly reflected in regulating lipid metabolism and maintaining energy balance of myocardial cells. It was found that the activity of PPARα was affected by the state of diabetes, and its expression and function might be inhibited, leading to lipid metabolism disorder and myocardial cell function damage (Lee et al., 2013). In addition, the activity of PPARα can also affect myocardial mitochondrial function, including mitochondrial morphology, quantity, respiratory chain complexes, Δψm, and oxidative stress response (Yin et al., 2019). Therefore, in-depth study on the expression and regulation mechanism of PPARα in diabetes cardiomyopathy and the relationship between PPARα and mitochondrial function will help reveal the molecular mechanism of the development of diabetes cardiomyopathy and provide new targets and strategies for disease treatment. Table 1 Summarizes the intervention measures and targets targeting mitochondrial dysfunction in the progress of DCM.

**TABLE 1:** The intervention measures and targets targeting mitochondrial dysfunction in the progress of diabetes cardiomyopathy.

Intervention	Model	Target spots or pathways	Effect	References
Melatonin	STZ injection mice	SIRT1/PGC-1α, Drp1	Inhibit mitochondrial fission, reduce oxidative stress	Ding et al. (2018)
Nicotinamide riboside	db/db mice	SIRT1/PGC-1α/PPARα, Mfn2	Promotes mitochondrial fusion	Hu et al. (2022)
MiR-144	STZ injection mice	AMPK /PGC-1α	Increases mitochondrial biogenesis, reduced cell apoptosis	Tao et al. (2020)
Adiponectin	db/ob mouse	AMPK/PGC-1α	Increases mitochondrial biogenesis	Yan et al. (2013)
Tetrahydrobiopterin	ob/ob mouse	CaMKK2/PGC-1α	Increase mitochondrial biogenesis	Kim et al. (2020)
Tat-Beclin1	High-fat diet -induced GFP-LC3 mouse	Parkin	Induce mitophagy	Tong et al. (2019)
Alisporivir	High-fat diet and STZ injection rats	PINK1, Parkin	Induce mitophagy	Belosludtseva et al. (2021)
Guanosine diphosphate	db/db mice	UCPs	Inhibit mitochondrial uncoupling	Dludla et al. (2018)
Grpel2	STZ injection mice	MCU, DLST	Inhibit mitochondrial calcium	Yang et al. (2023)
Mitsugumin 53	High-fat diet -induced obese mice	IRS-1	Improve insulin resistance	Liu et al. (2015)
Metformin	STZ injection mice	AMPK/PGC-1α, TFAM	Induce mitophagy, improve mitochondrial biogenesis	Liu et al. (2020), Abdel et al. (2018)
SGLT2is	High-fat diet and STZ injection rats	AMPK, PGC-1α, NRF, TFAM	Reduce oxidative stress,improve mitochondrial biogenesis	Li and Zhou (2020), Wiviott et al. (2019)

## 5 Plant secondary metabolites-based diabetes cardiomyopathy targeting mitochondrial dysfunction

**FIGURE 4:**
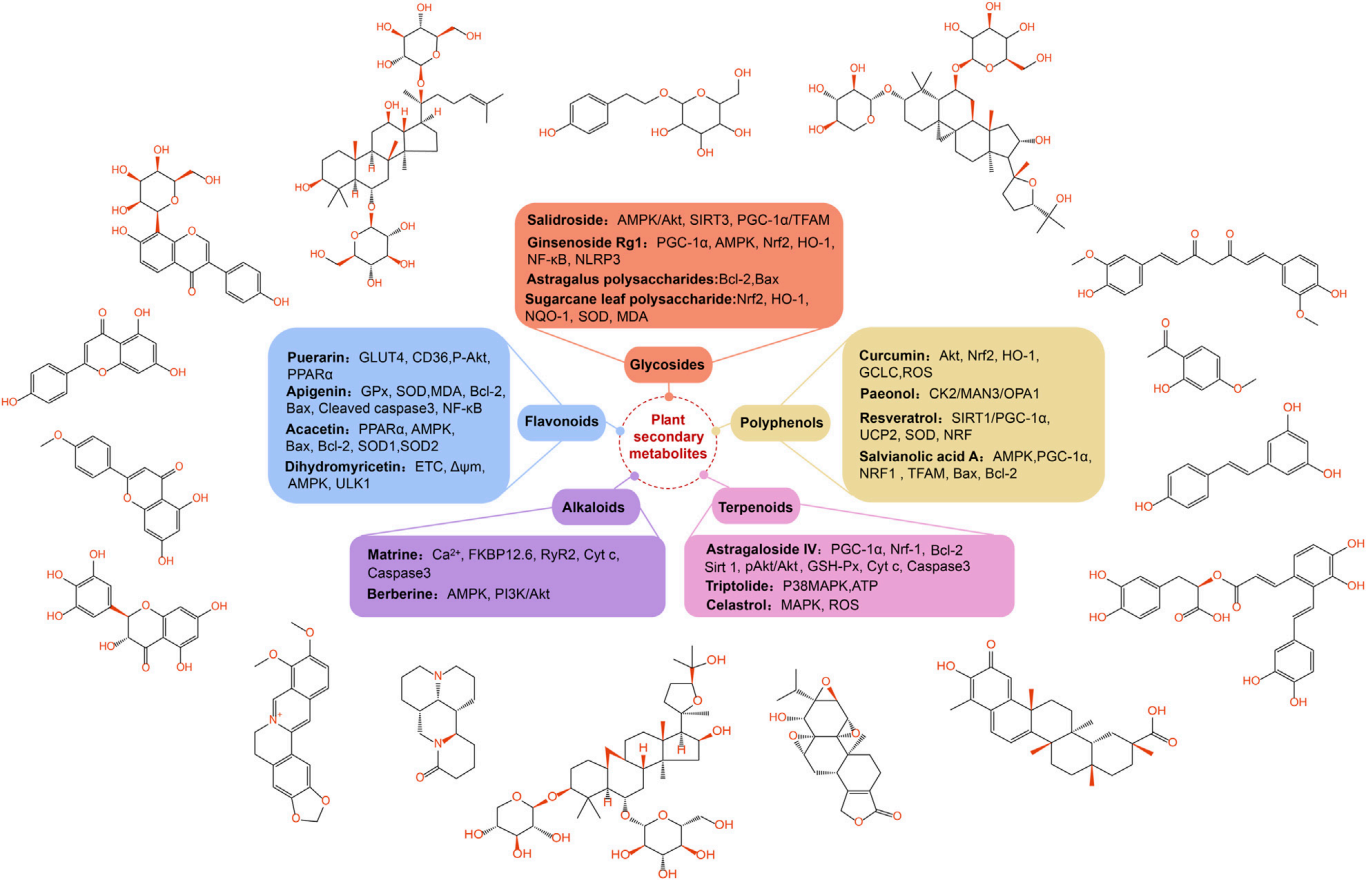
The classification of plant secondary metabolites and their main targets of regulating diabetes cardiomyopathy. Some plant secondary metabolites have been found to alleviate the pathological changes of diabetes cardiomyopathy, including flavonoids, polyphenols, terpenes, alkaloids and glycosides. These plant secondary metabolites are mainly regulated through targets related to mitochondrial function. Opa1, optic atrophy 1; UCP2, uncoupling protein 2; Nrf2, nuclear factor E2-related factor 2; HO-1, heme oxygenase-1; GClC, glutamate-cysteine ligase catalyst; MDA, malondialdehyde; NF-κB, nuclear factor kappa-B; ULK1, unc-51 like autophagy activating kinase 1; GSH, glutathione; GSH-Px, glutathione peroxidase; Nrf-1, nuclear respiratory factor-1; CK2α, casein kinase 2α; Stat3, signal transducer and activator of transcription 3; RyR2, Ryanodine receptor 2.

Secondary metabolites derived from plants have the properties of being safe, effective, and low in toxicity. Research on the prevention and treatment of diabetes and its complications using these metabolites has attracted increasing attention (Sukhikh et al., 2023). Previous studies have reported that the research on plant secondary metabolites for diabetes mainly focuses on regulating lipid and protein metabolism pathways, insulin signaling pathways, anti-inflammatory responses, and anti-oxidant stress (Shehadeh et al., 2021). In recent years, targeting mitochondrial function has become a promising treatment strategy for various diseases. Therefore, influencing mitochondrial function may have beneficial effects on DCM. This section reviews some biologically active plant secondary metabolites targeting mitochondrial dysfunction for the treatment of DCM (Figure 4).

### 5.1 Flavonoids

Flavonoids are secondary metabolites of plants, characterized by compounds with a 2-phenylchromen-4-one structure, widely present in plants. They exhibit various pharmacological activities, including antioxidant, anti-inflammatory, and anti-tumor effects, and have been used to treat various diseases (including diabetes) (Shen et al., 2022). Flavonoids are considered promising anti-diabetic drugs, but their poor bioavailability is also recognized. The use of drug delivery technologies such as microencapsulation, nano delivery systems, microemulsions, and enzyme-promoted methylation can enhance the therapeutic effects and bioavailability of flavonoids (Hussain et al., 2020). Flavonoids have a significant hypoglycemic effect by regulating the activity of mitochondrial respiratory chain complexes, affecting oxidative phosphorylation in mitochondria, reducing oxidative stress, improving mitochondrial energy metabolism and ATP synthesis, and helping to reduce the risk of diabetes and its complications (Sapian et al., 2021).

Puerarin is a flavonoid compound isolated from *Pueraria lobata* (Willd.) Ohwi, which has pharmacological activities such as reducing insulin resistance, alleviating inflammatory reactions, improving microcirculation, and inhibiting platelet aggregation (Huang et al., 2020). Cheng et al. found that after 4 weeks of treatment with puerarin (100 mg/kg/d), the expression and translocation of GLUT4 increased, while the expression and translocation of CD36 decreased in STZ and nicotinamide (NA)-induced diabetic mice. Puerarin also enhances Akt phosphorylation, reduces PPARα expression, and improves heart function after myocardial infarction in diabetes mice by regulating mitochondrial energy metabolism (Cheng et al., 2015). In a controlled trial involving 50 patients undergoing heart valve replacement, puerarin appears to enhance the safety and effectiveness of valve replacement surgery. Pretreatment with puerarin reduces the activation of neutrophil NF-κB and the overexpression of IL-6, IL-8, inhibits the release of cardiac enzymes troponin I (cTnI), and creatine kinase isoenzyme MB (CK-MB), indicating a protective effect on the myocardium (Zhou et al., 2019). Additionally, Sun et al.'s research has shown that Puerarin-V (a new crystal form of puerarin) can significantly reduce mitochondrial ROS production, decrease MDA levels, increase the activity of SOD and GSH in the myocardium, improve the activity of the mitochondrial electron transport chain, and enhance the mitochondrial respiratory function related to complexes I/II in DCM mice. These results indicate that puerarin-V plays an antioxidant role in DCM and may be involved in improving mitochondrial dysfunction. Furthermore, the therapeutic effect of puerarin-V in DCM is superior to that of puerarin injection (a marketed drug for myocardial ischemia), indicating that puerarin-V may be an attractive compound for developing anti-DCM drugs (Sun et al., 2022).

Apigenin is one of flavonoids widely found in plants, fruits, and vegetables, with anti-oxidant, anti-inflammatory, and anti-tumor effects (Salehi et al., 2019). Liu et al. research has confirmed that treatment with apigenin (100 mg/kg/d) for 7 months can significantly improve myocardial remodeling and cardiac function in STZ-induced diabetic C57BM/6J mice models. It also inhibits myocardial cell apoptosis, improves myocardial mitochondrial oxidative stress and inflammatory response, and normalizes myocardial mitochondrial energy. These pathological changes are achieved by inhibiting the excessive accumulation of 4-hydroxynonenal through apigenin, upregulating the expression of Bcl-2 and GPx, increasing SOD activity, reducing malondialdehyde (MDA) activity, downregulating the expression of Bax and Cleaved caspase3, and inhibiting the translocation of NF-κB (Liu et al., 2017). Additionally, apigenin has been found to reverse mitochondrial dysfunction induced by lipopolysaccharide (LPS), maintaining mitochondrial homeostasis and function by promoting the expression of mitochondrial SIRT3, inducing mitochondrial biogenesis (PGC-1α, TFAM) and fusion proteins (Mfn2, Opa1), and activating mitochondrial autophagy (PINK1, parkin) (Ahmedy et al., 2022). In conclusion, the results suggest that apigenin may be a promising compound for treating diabetic cardiac damage and neurological diseases by targeting mitochondrial function.

Acacetin is a common natural flavonoid compound that can be extracted and isolated from *Carthamus tinctorius* L. Pharmacological studies have shown that it has antioxidant, anti-tumor, anti-inflammatory, and cardiovascular protective effects (Han et al., 2021). The study by Song et al. demonstrated that in the STZ-induced Sprague-Dawley (SD) diabetic rat model, treatment with acacetin (10 mg/kg/d) for 16 weeks activated AMPK protein phosphorylation and regulated the expression levels of PPARα. *In vitro* experiments showed that acacetin (0.3, 1, 3 μM) downregulated the expression of Bax protein in H_9_C_2_ cells, while up-regulating the expression of Bcl-2, SOD1 (located in the intermembrane space of mitochondria), and SOD2 (mainly located in the mitochondrial matrix). The study suggests that acacetin can reduce oxidative stress, inhibit mitochondria-dependent cell apoptosis, improve mitochondrial function, and alleviate diabetic myocardial damage (Song et al., 2022). In addition, Han et al. found that acacetin can reduce ROS production and levels of MDA, inhibit depolarization of Δψm, upregulate the expression and activity of SOD, Bcl-2, PGC-1α, pAMPK, Sirt1, and Sirt3, and exert its cardioprotective effect. It is worth noting that when Sirt3 is knocked out, the cardioprotective effect of acacetin is eliminated (Han et al., 2020). In conclusion, research shows that acacetin can prevent mitochondrial dysfunction, reduce oxidative stress, and reduce the incidence of cardiovascular disease in diabetes.

Dihydromyricetin is a dihydroflavonol compound widely present in plants of the ampelopsis family, with pharmacological effects including scavenging free radicals, antioxidant, and anti-fibrotic properties (Zhang et al., 2022). Wu et al. found that treatment with dihydromyricetin (100 mg/kg/d) for 14 weeks improved mitochondrial function in STZ-induced diabetic C57BL/6J mice, increasing ATP content in, ETC., and the activity of complex I/II/III/IV/V, restoring Δψm, reducing oxidative stress, and improving mitochondrial energy metabolism. Furthermore, the study also indicated that dihydromyricetin can activate AMPK and the phosphorylation level of unc-51 like autophagy activating kinase 1 (ULK1), enhancing autophagic function in diabetic mice and preventing the occurrence of cardiac dysfunction (Wu et al., 2017). Hua et al. believe that dihydromyricetin may lower fasting blood glucose and glycated hemoglobin levels in diabetic mice, inhibit the production of ROS in mitochondria, upregulate SIRT3, SOD2 protein expression, and increase mtDNA copy number to suppress oxidative stress in diabetic mice and improve diabetic vascular endothelial dysfunction. This may be achieved through mediating SIRT3-dependent pathways (Hua et al., 2020). These research findings suggest that dihydromyricetin may have significant potential in regulating mitochondrial biosynthesis, stimulating mitochondrial autophagy, and combating oxidative stress.

### 5.2 Terpenoids

Terpenoids are polymers of isoprene and its derivatives, and they are very important secondary metabolites in plants. Terpenoids have shown anti diabetes properties *in vivo* and *in vitro* studies, which can increase insulin secretion in body tissues, promote the translocation of GLUT4 to increase glucose uptake, protect pancreatic cells and improve the expression of inflammatory factors (Putta et al., 2016). Recent reports indicate that terpenoids may also improve the development of diabetes cardiomyopathy by regulating mitochondrial function (Zhang et al., 2024).

Triptolide is a diterpenoid compound isolated from *Tripterygium wilfordii* Hook, possessing pharmacological effects such as anti-inflammatory, immune regulation, and anti-cancer properties (Gao et al., 2021). Liang et al. treated SD diabetic rats induced by STZ with triptolide (100, 200, or 400 μg/kg/d) for 6 weeks and evaluated cardiac energy metabolism using P-31 nuclear magnetic resonance spectroscopy. The study results indicated that the optimal therapeutic effect was achieved with a dose of 200 μg/kg/d of triptolide, which could enhance cardiac energy metabolism by promoting mitochondrial ATP generation and upregulating the expression of P38 MAPK protein, improving cardiac function in diabetic cardiomyopathy rats through the regulation of MAPK signaling pathways (Liang et al., 2015). Additionally, Pan et al.'s research indicates that during the process of cardiac remodeling, the expression of FoxP3 is downregulated in cardiomyocytes, leading to sustained activation of Parkin-mediated mitochondrial autophagy. However, Triptolide can regulate mitophagy, restoring the activity of FoxP3 in cardiomyocytes. Mechanistically, FoxP3 interacts with a sequence downstream of the binding site for Activating Transcription Factor 4 (ATF4), which involves the promoter of Parkin and sequestered free nuclear ATF4, to reduce the expression of Parkin mRNA during the process of cardiac remodeling. In conclusion, studies suggest that triptolide may be an effective cardioprotective agent (Pan et al., 2022).

Celastrol is one of terpenoids isolated from *T. wilfordii* Hook, which has various biological activities such as anti rheumatoid, anti-tumor, and antioxidant properties (Wang et al., 2020). Wu et al. used network pharmacology to predict the key regulatory targets of celastrol on DCM, and analyzed its biological processes and signaling pathways through animal experiments. The results showed that celastrol (50 μg/kg/d) treatment for 4 weeks could downregulate the expression of P38 protein in the MAPK pathway, reverse the energy remodeling, mitochondrial dysfunction, and oxidative stress induced by STZ-induced SD diabetic rats, thereby delaying the deterioration of heart function and myocardial interstitial fibrosis. The study suggests that the MAPK signaling pathway may be an effective intervention target for DCM (Wu et al., 2022). In another study, celastrol can also alleviate diabetes-induced cardiac damage, inhibit mitochondrial ROS production, and suppress the release of inflammatory factors. The research results indicate that celastrol shows great potential as an effective cardiac protective drug for treating DCM (Zhao et al., 2023).

Astragaloside IV is one of the active ingredients extracted from *Astragalus membranaceus* (Fisch.) Bunge, which has pharmacological effects such as anti-inflammatory, antioxidant, immunomodulatory, and anti-tumor effects (Gao et al., 2022). In a SD rat model of DCM induced by STZ, Zhang et al. confirmed that astragaloside IV (10, 20, and 40 mg/kg/d) treatment for 16 weeks can improve mitochondrial biogenesis by upregulating the expression of PGC-1α and Nrf-1 in myocardial tissue, as well as PGC-1α and Nrf-1 mRNA expression in H_9_C_2_ cells. This regulation enhances ATP and ADP levels to improve mitochondrial energy metabolism, reduces the expression of cytochrome c (Cyt c) and caspase-3 to inhibit cell apoptosis and myocardial hypertrophy, thereby reducing diabetic myocardial damage (Zhang et al., 2019). Additionally, Zhu et al. have shown that astragaloside IV can downregulate the expression of miR-34a and upregulate the expression of Bcl-2, Sirt1, and pAkt/Akt proteins to protect myocardial cells from high glucose-induced damage (Zhu et al., 2019). These findings suggest that astragaloside IV may exert a protective effect on DCM by promoting mitochondrial biogenesis and inducing mitophagy.

### 5.3 Polyphenols

Polyphenols refer to secondary metabolites in plants, named for their multiple phenolic groups. They are widely present in traditional herbal medicines and some natural foods. In the treatment of DCM, polyphenols demonstrate significant antioxidant activity, capable of scavenging free radicals, reducing oxidative stress, and thus protecting mitochondria from oxidative damage (Raina et al., 2024).

Curcumin is a polyphenolic compound isolated from the root of *Curcuma longa* L and extensive research has confirmed that it is a highly effective antioxidant (Zheng et al., 2018). In a rat model of SD diabetes established by HFD and intraperitoneal injection of STZ, curcumin (200 mg/kg/d) treatment for 8 months promoted the transfer of Nrf2 to the nucleus through the AKT pathway, increased the expression of the antioxidant factors HO-1 and GCLC, reduced the accumulation of mitochondrial ROS, and mitigated mitochondrial oxidative damage. The study suggests that curcumin inhibits cell apoptosis by activating the AKT/Nrf2/ARE pathway and eliminates the accumulation of superoxide in myocardial cells (Wei et al., 2023). Additionally, Yao et al. research has shown that curcumin can upregulate the expression of AMPK and JNK1 to stimulate mitophagy, as well as upregulate the expression of Bcl-2 and Bim to reduce cardiomyocyte apoptosis. Further mechanistic studies have indicated that curcumin prevents DCM through the cross-talk between mitophagy and apoptosis mechanisms via the AMPK/mTORC1 pathway (Yao et al., 2018).

Paeonol is a polyphenolic compound isolated from the root bark of *Paeonia suffruticosa* Andr, and it is also the active ingredient of paeonol injection (a marketed antipyretic and analgesic drug), which has pharmacological effects such as anti-inflammatory, neuroprotective, and anti-cardiovascular diseases (Zhang et al., 2019). The research by Liu et al. found that intervention with paeonol (75, 150, or 300 mg/kg/d) for 12 weeks in STZ-induced diabetic rats promoted Opa1-mediated mitochondrial fusion, inhibited mitochondrial oxidative stress, and maintained mitochondrial respiratory capacity and cardiac function in DCM. It is noteworthy that knocking out Opa1 attenuated the protective effect of paeonol in diabetic hearts and high-glucose-treated cardiomyocytes. The study indicates that paeonol is a novel promoter of mitochondrial fusion, providing protection against DCM through the CK2-MAN3-OPA1 signaling pathway (Liu et al., 2021). Additionally, in another study, Ding et al. demonstrated that paeonol can activate the transcription factor Stat3 to promote Mfn2-mediated mitochondrial fusion, not only reducing doxorubicin (Dox)-induced cardiac toxicity, but also preserving Dox’s anticancer activity (Ding et al., 2023). In conclusion, these research findings suggest that paeonol may have significant value in preventing or treating diabetes and its complications by regulating mitochondrial dynamics.

Resveratrol is a polyphenolic compound widely present in plants, fruits, and vegetables, with biological activities such as antioxidant, anti-inflammatory, anticancer, and anti-aging (Galiniak et al., 2019). Fang et al. believe that resveratrol (50 mg/kg/d) treatment for 16 weeks significantly alleviated the cardiac dysfunction induced by HFD combined with STZ in SD rats. This was manifested by a significant increase in the activity of manganese SOD, ATP content, mitochondrial DNA copy number, Δψm, and nuclear respiratory factor (NRF), and a significant decrease in MDA and mitochondrial uncoupling protein UCP2 levels. The results indicate that resveratrol alleviates cardiac dysfunction in diabetic rats by improving mitochondrial function through SIRT1-mediated PGC-1α deacetylation (Fang et al., 2018). Similarly, in another study, Diao et al. demonstrated that resveratrol treatment improved mitochondrial function in diabetic rats, inhibited mitochondrial ROS generation, MPTP opening, and Cyto c release. It also suppressed the expression of UCP2 protein, thereby improving cardiac function in diabetic rats (Diao et al., 2019). In conclusion, resveratrol may have a positive impact on the prevention and treatment of diabetic cardiomyopathy by regulating mitochondrial uncoupling.

Salvianolic acid A is a polyphenolic compound isolated from *Salvia miltiorrhiza* Bunge, which has been proven to have various biological activities, including anti-oxidant, anti-inflammatory, anti-fibrotic and neuroprotective, etc (Wang et al., 2019). The research by Gong et al. indicates that Salvianolic acid A (3 mg/kg/d) treatment for 6 weeks in STZ-induced diabetic SD rats significantly enhances the respiratory activity and mitochondrial respiratory function related to complex I/II in diabetic rats, improves the abnormal electrocardiogram in diabetic rats, and inhibits cardiomyocyte apoptosis by down-regulating the expression of Bax and up-regulating the expression of Bcl-2, Caspase3, and Caspase9, thus exerting a protective effect on the heart (Gong et al., 2023). Furthermore, Wang et al.'s research indicates that salvianolic acid A can promote mitochondrial biogenesis in endothelial cells by regulating the expression of AMPK, PGC-1α, NRF1, and TFAM. Mechanistically, salvianolic acid A may activate the AMPK-mediated PGC-1α/TFAM signaling pathway, thereby improving the occurrence of diabetic cardiovascular diseases caused by mitochondrial dysfunction (Wang et al., 2022). In conclusion, Salvianolic acid A can prevent and treat diabetic cardiomyopathy by enhancing mitochondrial respiratory function and promoting mitochondrial biogenesis.

### 5.4 Alkaloids

Alkaloids are plant secondary metabolites composed of polycyclic aromatic frameworks containing one or more nitrogen atoms. Alkaloids have significant hypoglycemic effects, as they can stimulate glucose uptake and regulate insulin secretion, and are considered allosteric activators of AMPK (Seksaria et al., 2023). It is reported that alkaloids can affect the permeability of mitochondrial membrane, regulate the level of calcium ions in mitochondria, and regulate the imbalance of mitochondrial dynamics, thus reducing the occurrence of mitochondrial dysfunction, which is beneficial to the treatment of DCM (Patalas et al., 2021).

Berberine is an isoquinoline alkaloid that can be extracted and isolated from various plants such as *Coptis chinensis* Franch and *Phellodendron chinense* Schneid. It has biological activities such as lowering blood sugar and regulating lipid metabolism (Pang et al., 2015). In a study by Hang et al., it was shown that in a high glucose-induced H_9_C_2_ cardiomyocyte hypertrophy model, berberine intervention for 24 h at a concentration of 100 nM can regulate the imbalance of mitochondrial dynamics, promote mitochondrial biogenesis, and activate mitophagy to eliminate damaged mitochondria. These beneficial effects of berberine may be related to the activation of the AMPK signaling pathway (Hang et al., 2018). Additionally, research by Chen et al. demonstrated that berberine can upregulate the Bcl-2/Bax ratio, reduce the expression of Caspase3 protein, and simultaneously activate the PI3K/Akt and AMPK signaling pathways to improve cardiac contractile and diastolic dysfunction in diabetic rat myocardial I/R and inhibit myocardial cell apoptosis (Chen et al., 2014). Therefore, berberine may treat DCM by targeting the AMPK and PI3K/Akt signaling pathways through the activation of mitochondrial biogenesis.

Matrine is an alkaloid extracted and isolated from the dried roots of *Sophora flavescens* Aiton. It exhibits a wide range of biological activities, such as antioxidant, anti-tumor, anti-inflammatory, anti-fibrotic, anti-arrhythmic, and immunomodulatory effects (Wang et al., 2023). In an AGEs-induced SD rat model, matrine (50, 100, and 200 mg/kg/d) treatment for 20 days was found to inhibit the dissociation of FKBP12.6 and RyR2, reduce RyR2 activity and Ca^2+^ levels, decrease the expression levels of cytochrome c and active Caspase3, suppress cell apoptosis, and restore Δψm (Wang et al., 2019). Additionally, studies by Liu et al. suggest that matrine significantly reduces mitochondrial ROS production in primary cardiomyocytes of DCM rats, downregulates the expression of Cleaved caspase8 and Cleaved caspase3 proteins to inhibit cardiomyocyte apoptosis. Further research indicates that matrine improves diabetic cardiomyopathy by inhibiting the ROS/TLR-4 signaling pathway (Liu et al., 2015). In conclusion, matrine can effectively improve the occurrence of diabetic cardiac dysfunction and may potentially be developed as a cardioprotective agent.

### 5.5 Glycosides

Glycosides are an important type of plant secondary metabolites, widely found in plants, fruits, vegetables, and nuts. They include saponin glycosides, flavonoid glycosides, alcoholic glycosides. Phenolic glycosides, coumarin glycosides, and more. Glycosides hold great potential in the prevention and treatment of diabetes and various vascular complications (Yeram et al., 2022).

Ginsenoside Rg1 is a saponin glycoside isolated from *Panax ginseng* C. A. Mey, which has various biological activities such as anti-inflammatory, anti-oxidant, anti-platelet aggregation, anti-cancer, hypoglycemic, and neuroprotective effects (Zhang et al., 2022). Qin et al. showed that ginsenoside Rg1 (20 mg/kg/d) can promote mitochondrial biogenesis by increasing the expression of PGC-1α, AMPK, Nrf2 and HO-1 proteins, and reduce the expression of NF-κB and NLRP3 proteins to reduce oxidative stress after 8 weeks of treatment in STZ induced diabetes Wistar rats. Furthermore, ginsenoside Rg1 has been found to play a cardioprotective role by mediating the mitochondrial-related AMPK/Nrf2/HO-1 signaling pathway (Qin et al., 2019). In another study, ginsenoside Rg1 significantly reduced MDA and caspase-3 levels in the myocardium of diabetic rats, while increasing levels of SOD, catalase, glutathione peroxidase (GSH-Px), and B-cell lymphoma-extra-large (Bcl-xL). This indicates that ginsenoside Rg1’s treatment of diabetic rats is associated with inhibiting oxidative stress and alleviating myocardial cell apoptosis (Yu et al., 2015). In conclusion, these findings suggest that ginsenoside Rg1 may have potential preventive and therapeutic effects on cardiovascular damage in diabetic patients by regulating mitochondrial biogenesis, inhibiting oxidative stress, and the mitochondrial-dependent apoptotic pathway.

Salidroside is an alcohol glycoside isolated from *Rhodiola rosea* L, which has a wide range of biological activities, including antioxidant, anti-tumor, antiviral, and hypoglycemic effects (Rong et al., 2020). The research by Li et al. indicates that in a C57BLKS/J mice model induced by HFD and STZ injection, treatment with salidroside (50 or 100 mg/kg/d) for 16 weeks can improve insulin resistance, mitochondrial ultrastructure damage, and restore normal cardiac contractile function in diabetic mice. Further mechanistic studies have shown that salidroside upregulates the expression of SIRT3 protein, promotes the translocation of SIRT3 from the cytoplasm to the mitochondria, increases the deacetylation of mitochondrial protein MnSOD, and upregulates the expression of AMPK, PGC-1α, and TFAM to induce mitochondrial biogenesis (Li et al., 2021). Additionally, salidroside can improve DCM by activating the Akt signaling pathway, upregulating the expression of Nrf2 and the antioxidant factor HO-1 (Ni et al., 2021). In conclusion, salidroside may play an important role in diabetes and its cardiovascular complications by promoting mitochondrial biogenesis and exerting antioxidant stress.

Astragalus polysaccharides are water-soluble polysaccharides extracted from the dried roots of *Astragalus membranaceus* (Fisch.) Bunge, which have anti-oxidant, anti-inflammatory, anti diabetes, immune regulation and other biological activities (Dong et al., 2023). Studies by Sun et al. have shown that astragalus polysaccharides (0.1–3.2 mg/mL, 24 h) can inhibit HG-induced H_9_C_2_ cell apoptosis by up-regulating the expression of Bcl-2, down-regulating the expression of Bax, and increasing the ratio of Bcl-2/Bax in the mitochondria (Sun et al., 2017). In another study, Chen et al. demonstrated that astragalus polysaccharides can protect the ultrastructure of cell mitochondria, reduce cell apoptosis, increase SOD activity, and thereby reduce oxidative stress induced by HG in H_9_C_2_ cells (Chen et al., 2018). In conclusion, these results prove that astragalus polysaccharides can prevent and treat DCM through the mitochondria-mediated apoptotic pathway.

Sugarcane leaf polysaccharide is an amorphous polysaccharides isolated from *Saccharum sinensis* Roxb leaves, which has various biological activities such as antioxidant, hypoglycemic, lipid-lowering, antibacterial, and immune regulation (Tang et al., 2019). Studies by Sun et al. have shown that sugarcane leaf polysaccharide (10 and 20 mg/kg/d) can effectively reverse myocardial ischemia-reperfusion injury in diabetic rats, prevent myocardial fibrosis and neutrophil infiltration, increase myocardial tissue SOD activity, reduce MDA and MPO activity, and significantly inhibit the expression levels of TNF-α and IL-6. *In vitro*, it promotes the translocation of Nrf2 from the cytoplasm to the nucleus by activating the Nrf2/HO-1 signaling pathway, upregulates the expression of Nrf2, HO-1, and NQO-1 proteins, reduces ROS production, and restores Δψm to affect myocardial mitochondrial biogenesis (Sun et al., 2023). In addition, Hao et al. suggested that sugarcane leaf polysaccharides can promote the expression of vascular endothelial growth factor (VEGF), enhance the activity of SOD, reduce the levels of MDA, NO, and GSH-Px, strengthen the antioxidant capacity in NOD mice, facilitate the body in clearing oxidative free radicals, and thereby improve the oxidative stress status of pancreatic β-cells (Hao et al., 2018). These research results indicate that sugarcane leaf polysaccharide may prevent DCM through targeting mitochondrial biogenesis and enhancing antioxidant capacity. Table 2 Plant secondary metabolites targeting mitochondrial dysfunction in diabetes cardiomyopathy models.

**TABLE 2:** Plant secondary metabolites targeting mitochondrial dysfunction in diabetes cardiomyopathy models.

Category	Metabolites name	Molecular formula	In vivo model	In vitro model	Effect	Possible mechanisms/ target	References
Flavonoids	Puerarin	C_21_H_20_O_9_	STZ-NA induced C57BL/6J male mice (100 mg/kg/d, 4 weeks)	—	Improve insulin resistance, regulate mitochondrial respiratory function	GLUT4, CD36,P-Akt, PPARα	Huang et al. (2020)
	Apigenin	C_15_H_10_O_5_	STZ induced C57BL/6J male mice(100 mg/kg/d, 7 months)	High glucose induced H_9_C_2_ cell (25 μM, 12, 24, 72 h)	Induce mitochondrial biogenesis, promote mitochondrial fusion	GPx, SOD,MDA, Cleaved caspase3, Bcl-2, Bax, NF-κB	Liu et al. (2017)
	Acacetin	C_16_H_12_O_5_	STZ induced SD rats (10 mg/kg/d, 16 weeks)	High glucose induced H_9_C_2_ cell(0.3, 1, 3 μM, 48 h)	Reduce oxidative stress, inhibit mitochondrial dependent cell apoptosis	PPARα, AMPK, Bax, Bcl-2, SOD1, SOD2	Song et al. (2022)
	Dihydromyricetin	C_15_H_12_O_8_	STZ induced C57BL/6J male mice(100 mg/kg/d, 14 weeks)	—	Reduce oxidative stress, regulate mitochondrial energy metabolism	ETC, Δψm, AMPK, ULK1	Wu et al. (2017)
Terpenoids	Triptolide	C_20_H_24_O_6_	STZ induced SD rats (100, 200, and 400 μg/kg/d, 6 weeks)	—	Improve insulin resistance, regulate mitochondrial energy metabolism	P38MAPK, ATP	Liang et al. (201)
	Celastrol	C_29_H_38_O_4_	STZ induced SD rats(50 μg/kg/d, 4 weeks)	—	Improve insulin resistance, reduce oxidative stress	MAPK, ROS	Wu et al. (2022)
	Astragaloside IV	C_41_H_68_O_14_	STZ induced SD rats (10, 20 and 40 mg/kg/d, 16 weeks)	High glucose induced H_9_C_2_ cell (20, 40 and 80 µmol/L, 48 h)	Promote mitochondrial biogenesis, induce mitophagy	PGC-1α, Nrf-1, MDA, GSH, PI3K/Akt, GSH-Px, Cyt c, Caspase3	Zhang et al. (2019)
Polyphenols	Curcumin	C_21_H_20_O_6_	High-fat diet and STZ induced SD rats (200 mg/kg/d, 8 months)	High glucose induced primary rat cardiomyocytes (14 μm/L, 24 h)	Reduce oxidative stress, induce mitophagy	Akt, Nrf2, HO-1, GCLC, ROS	Wei et al. (2023)
	Paeonol	C_9_H_10_O_3_	STZ induced SD rats (75, 150, and 300 mg/kg/d, 12 weeks)	High glucose induced primary rat cardiomyocytes (25, 50, 100 and 200 μmol/L, 48 h)	Promote mitochondrial fusion,reduce oxidative stress	CK2/MAN3/OPA1	Liu et al. (2021)
	Resveratrol	C_14_H_12_O_3_	High-fat diet and STZ induced SD rats (50 mg/kg/d, 16 weeks)	—	Inhibite mitochondrial uncoupling	SIRT1/PGC-1α, UCP2, SOD, NRF	Fang et al. (2018)
	Salvianolic acid A	C_26_H_22_O_10_	STZ induced SD rats (3 mg/kg/d, 6 weeks)	—	Promote mitochondrial biogenesis, inhibit mitochondrial dependent cell apoptosis	AMPK, PGC-1α, NRF1, TFAM, Bax, Bcl-2	Gong et al. (2023)
Alkaloids	Berberine	C_20_H_18_NO_4_	—	High glucose induced H_9_C_2_ cell line (100 nM, 24 h)	Promote mitochondrial biogenesis,induce mitophagy	AMPK, PI3K/Akt	Hang et al. (2018)
	Matrine	C_15_H_24_N_2_O	AGEs induced SD rats (50, 100, and 200 mg/kg/d, 20 days)	High glucose induced primary rat cardiomyocytes (0.5, 1.0 and 2.0 mmol/L, 24 h)	Regulate mitochondrial calcium handling	Ca^2+^, FKBP12.6, RyR2, Cyt c, Caspase3	Wang et al. (2019)
Glycosides	Ginsenoside Rg1	C_42_H_72_O_14_	STZ induced Wistar rats (20 mg/kg/d, 8 weeks)	—	Promote mitochondrial biogenesis	PGC-1α, AMPK, Nrf2, HO-1, NF-κB, NLRP3	Qin et al. (2019)
	Salidroside	C_14_H_20_O_7_	High fat diet and STZ induced C57BLKS/J mice (50 or 100 mg/kg/d, 16 weeks)	Primary culture of neonatal ratcardiomyocytes(10, 30 μM, 48 h)	Promote mitochondrial biogenesis	AMPK/Akt,SIRT3, PGC-1α/TFAM,MnSOD	Li et al. (2021)
	Astragalus polysaccharides	C_41_H_68_O_14_	—	High glucose induced H_9_C_2_ cell line(0.1, 0.2, 0.4, 0.8, 1.6 and 3.2 mg/mL, 24 h)	Inhibit mitochondrial dependent cell apoptosis	Bcl-2, Bax	Sun et al. (2017)
	Sugarcane leaf polysaccharide	—	Myocardial ischemia-reperfusion(MI/R) SD rat model (10 and 20 mg/kg/d, 1 weeks)	TBHP induced H_9_C_2_ cells (25, 50, and 100 μg/mL, 3 h)	Promote mitochondrial biogenesis	Nrf2, HO-1, NQO-1, ROS	Sun et al. (2023)

## 6 Traditional plants with hypoglycemic and antioxidant properties can regulate mitochondrial function in diabetic cardiomyopathy

Astragali radix (leguminous), huangqi in Chinese, also known as astragalus, the dried root of *Astragalus membranaceus* (Fisch.) Bge. var. mongholicus (Bge.) Hsiao or *Astragalus membranaceus* (Fisch.)Bge., is used as a traditional medicinal plants in China, Iran, Russia, and some other European countries. Astragali radix was first recorded in the Shennong’s Classic of Materia Medica and is listed as a qi-supplementing formula. In the theoretical system of traditional Chinese medicine (TCM), astragali radix is sweet in flavor, warm in nature, and acts on the lung and spleen. It has the traditional effects of invigorating qi for ascending, consolidating superficies for arresting sweating and inducing diuresis for removing edema (Liu et al., 2023). Currently, a variety of secondary metabolites have been isolated from the dried roots of astragali radix, including polysaccharides, flavonoids, saponins, amino acids, trace elements, etc. Among them, polysaccharides, flavonoids, and saponins have biological activities such as hypoglycemic, antioxidation, and immune regulation, and are important components of the pharmacological effects of astragali radix. In addition, pharmacological studies have shown that the biological activities of astragali radix, such as antioxidation, hypoglycemic, immune regulation, and antiinflammatory effects, are widely used in the treatment of respiratory, digestive, urinary, and blood system diseases, as well as diabetes and its complications, and have achieved good therapeutic effects (Chen et al., 2020). In previous reports, it has been indicated that astragaloside IV can significantly delay the excessive generation of mitochondrial ROS. It can also exert a protective effect on diabetic cardiomyopathy by upregulating the activities of antioxidant enzymes SOD2, catalase, GSH-Px, and downregulating the expression of c-Jun N-terminal kinase and p38 MAPK (Chen et al., 2018). Astragalus polysaccharides can prevent the occurrence of diabetic cardiomyopathy through the mitochondrial-mediated cell apoptosis pathway (Sun et al., 2017).

Kudzu root (*P. lobata* (Willd.)Ohwi) belongs to the leguminosae and is a homologous medicinal and edible plant. It is mainly distributed in southern China and Southeast Asia. Kudzu root is sweet in flavor, mild in nature, and acts on the spleen, stomach, and lung. It has traditional effects such as expelling pathogenic factors from muscles for clearing heat, relieving rigidity of muscles and activating collaterals. In clinical applications of TCM, kudzu root is often used to treat diabetes, cardiovascular and cerebrovascular diseases, tumors, and other ailments. Kudzu root has various pharmacological effects such as antioxidant, hypoglycemic, anti-inflammatory, anti-tumor, blood pressure lowering, lipid-lowering, heart protection, and memory improvement (Wang et al., 2022). The extract of kudzu root contains abundant flavonoids such as puerarin, daidzein, tectoridin, and luteolin-6-C-glucoside, which have free radical scavenging and antioxidant activities. Its mechanism of action may be related to the regulation of oxidative stress-related factors such as COX-2, SOD, MDA, ET-1, NO, and GSH (Gao et al., 2016; Dong et al., 2024). In DCM, puerarin has been found to modulate mitochondrial function. Studies have shown that puerarin can regulate mitochondrial energy metabolism, reduce oxidative stress and apoptosis of myocardial cells, and improve the symptoms of DCM (Sun et al., 2022). In general, kudzu root is a medicinal plant with wide-ranging biological activities that can improve diabetic heart function by modulating mitochondrial function and other pathways.

Carthami flos (asteraceae), the dried floret of *C. tinctorius* L., is a perennial herbaceous plant with rich medicinal value, mainly distributed in Iran, North Korea, China, Mongolia, Russia and other regions. In clinical applications of TCM, carthami flos is pungent in flavor, warm in nature, and acts on the heart and liver. It is traditionally believed to promote blood circulation for removing blood stasis, regulate qi-flowing for relieving pain, and is mainly used for angina, irregular menstruation, diabetes, and hypertension (Tu et al., 2015). Carthami flos contains compounds such as flavonoids, volatile oils, and polysaccharides, which have biological activities such as antioxidant and anti-inflammatory effects. Hydroxysafflor yellow A isolated from carthami flos is considered a potential antioxidant, providing protective effects against myocardial damage. Hydroxysafflor yellow A can increase the levels of SOD and GPX 1 in the serum of DCM mice, reduce MDA content, scavenge free radicals, and reduce oxidative stress damage to cardiac mitochondria (Yao et al., 2021). In addition, the essential oil of carthami flos extracted using different solvents was analyzed by gas chromatography-mass spectrometry (GC-MS) technology. The content of n-hexane extract was 97.65%, petroleum ether extract was 98.05%, dichloromethane extract was 98.93%, and the content of steam-distilled extract was 99.68%. *In vitro* pharmacology studies have shown that the n-hexane extract of carthami flos has the best *in vitro* anti-diabetic activity against protein tyrosine phosphatase 1B (PTP1B), demonstrating potential for the treatment of diabetes and obesity (Li et al., 2012).

*Gynostemma pentaphyllum* (Thunb.) Makino is a kind of cucurbitaceae, mainly distributed in India, Nepal, Bangladesh, China, Myanmar, Laos, Vietnam, Malaysia and other regions. Gynostemma pentaphyllum is spicy and slightly bitter in flavor, warm in nature, and acts on the lung, spleen, and stomach. In the theory of TCM, it has the traditional effects of warming spleen and stomach for dispelling cold, ventilating lung qi for dissipating phlegm and regulating qi-flowing for harmonizing stomach. Gynostemma pentaphyllum is a commonly used plant in TCM for treating diabetes. It has various pharmacological effects such as antioxidant, hypoglycemic, anti-inflammatory, antibacterial, antiallergic, and antitumor properties (Nguyen et al., 2021). Active ingredients in gymnema pentaphyllum, such as saponins and polysaccharides, have a certain hypoglycemic effect, significantly reducing insulin resistance index and improving diabetes and its complications. Gypenoside can lower fasting blood sugar and blood lipids in mice with type 2 diabetes induced by HFD and STZ, and significantly improve glucose tolerance and insulin resistance. Its hypoglycemic effect may be related to the downregulation of key proteins in the AMPK signaling pathway, including phosphoinositide 3-kinase and glucose-6-phosphatase (Song et al., 2022). Similarly, in another study, the extract of gynostemma pentaphyllum significantly reduced the levels of MDA, hydrogen peroxide, peroxynitrite, and ROS in DCM rats, while increasing the levels of GSH, SOD, CAT, and GPx. It also significantly reduced the expression of cytokines and inflammatory parameters (TNF-α, IL-6, IL-1β, COX-2, NLRP3, NF-κB). Furthermore, the extract of gynostemma pentaphyllum also promoted mitochondrial biogenesis in cardiac tissues by enhancing the expression of PGC-1, HO-1, and Nrf2. These results indicate that the gynostemma pentaphyllum has a cardioprotective effect on STZ-induced diabetic cardiac dysfunction by regulating the AMPK/Nrf2/HO-1 pathway (Chen et al., 2022).

## 7 Conclusion

Diabetic cardiomyopathy is manifested as abnormal cardiac structure and function in the absence of ischaemic or hypertensive heart disease in individuals with diabetes. However, its pathogenesis remains unclear. Mitochondrial dysfunction is an important pathological mechanism leading to the development of the disease. Targeted regulation of mitochondrial function can effectively improve the symptoms of DCM. Targeting mitochondria with plant secondary metabolites may be an effective approach for preventing and treating DCM. This review provides evidence supporting mitochondrial dysfunction in DCM, briefly describes the pathophysiological mechanisms leading to mitochondrial dysfunction, and discusses potential targets and treatment strategies.

Currently, extensive screening research on anti-diabetic drugs has identified plants as the main potential source for drug discovery. Biologically active secondary metabolites in plants, such as flavonoids, terpenes, polyphenols, alkaloids, and glycosides, have been proven to have hypoglycemic effects *in vivo* and *in vitro*. Previous reports indicate that plant secondary metabolites improve hyperglycemia and insulin resistance mainly by regulating lipid and protein metabolism pathways, insulin signaling pathways, anti-inflammatory responses, and antioxidant stress. The key regulatory targets involved include α-glucosidase, α-amylase, dipeptidyl peptidase 4 (DPP-4), protein tyrosine phosphatase 1B (PTP1B), PPARα, GLUT4, and the AMPK signaling pathway (Shehadeh et al., 2021; Sukhikh et al., 2023).

In this review, we have gathered plant secondary metabolites that affect mitochondrial function in the treatment of DCM. Some plant secondary metabolites have biological activities such as hypoglycemic, anti-oxidation, and anti-inflammation, which can protect myocardial cells by improving mitochondrial dysfunction. For example, apigenin can promote mitochondrial biogenesis, induce mitochondrial autophagy to maintain the normal myocardial mitochondrial quality and quantity homeostasis; triptolide can improve insulin resistance, regulate mitochondrial energy metabolism; paeonol can promote mitochondrial fusion, inhibit mitochondrial oxidative stress; resveratrol can regulate the opening of mitochondrial inner membrane channels, regulate the process of mitochondrial uncoupling, and help reduce oxidative stress reactions inside mitochondria; matrine can regulate the opening of mitochondrial calcium ion channels, affecting the balance of calcium ions inside mitochondria; sugarcane leaf polysaccharide can improve insulin resistance and promote mitochondrial biogenesis. However, there are still some challenges in the use of plant secondary metabolites in the treatment of DCM. For example, the pharmacological effects and dosages of plant secondary metabolites are not fully understood, and a more detailed quality evaluation system is needed to verify their efficacy and safety. It is also important to address how to improve the bioavailability and stability of plant secondary metabolites, which can be achieved through nanocarrier delivery technology, chemical modification, and other biotechnological methods. In conclusion, plant secondary metabolites targeted at mitochondria are expected to become an important drug resource for the treatment of DCM, and more clinical experiments are needed in the future to elucidate their mechanisms of action.
